# Supplementary material for: Identification of evolutionarily conserved regulators of muscle mitochondrial network organization
Source: Nat Commun. 2022 Nov 4;13:6622. doi: 10.1038/s41467-022-34445-9 (PMC9636386; doi:10.1038/s41467-022-34445-9)
Supplement: Supplementary file 1 — Supplementary Information [file 41467_2022_34445_MOESM1_ESM.pdf]

Identification of Evolutionarily Conserved Regulators of Muscle Mitochondrial Network Organization

Separating Contractile and Mitochondrial Fiber Type

		Flight	Jump	Leg	*salm KD-* Flight	*salm OE-* Leg
Contractile type	Fibrillar	+	-	-	-	+
	Tubular	-	+	+	+	-
Mito Network type	Parallel	++	++	+	-	+
	Grid	-	-	+	+	+
	Salm	++	+	-	-	+

Supplementary Table 1. Combinations of muscle contractile type, mitochondrial network configuration and *salm* expression among the five different muscle types.

H15 OFF target (gene name)	Phenotype with Mef2 Gal4	Stock ID	Reference
**CG8127**	Weak Flyer	BS# 35780, BS# 43231	This Paper
**CG16711**	wild type		PMID: 20220848
**CG32717**	wild type	BS# 33909, BS# 33991	This Paper
**CG7847**	Lethal and weak flyer	BS# 27701	PMID: 20220848 and this Paper
**CG7031**	wild type		PMID: 20220848
**CG43122**	wild type	BS# 25995	PMID: 20220848, and This Paper
**CG43749**	wild type		PMID: 20220848
**CG11202**	Wildtype	BS# 62953	This Paper
**CG13194**	Weak Flyer	BS# 63547	This Paper
**CG33988**	wild type		PMID: 20220848
**CG11711**	Wild type		PMID: 20220848
**CG31374**	Lethal and Flight less	BS# 31548,	This Paper
**CG10192**	wild type		PMID: 20220848
**CG34380**	wild type		PMID: 20220848
**CG6175**	weak flyer	BS# 62516	PMID: 20220848 and This Paper
**CG12443**	Weak flyer		PMID: 20220848

Supplementary Table 2. List of off-target genes.

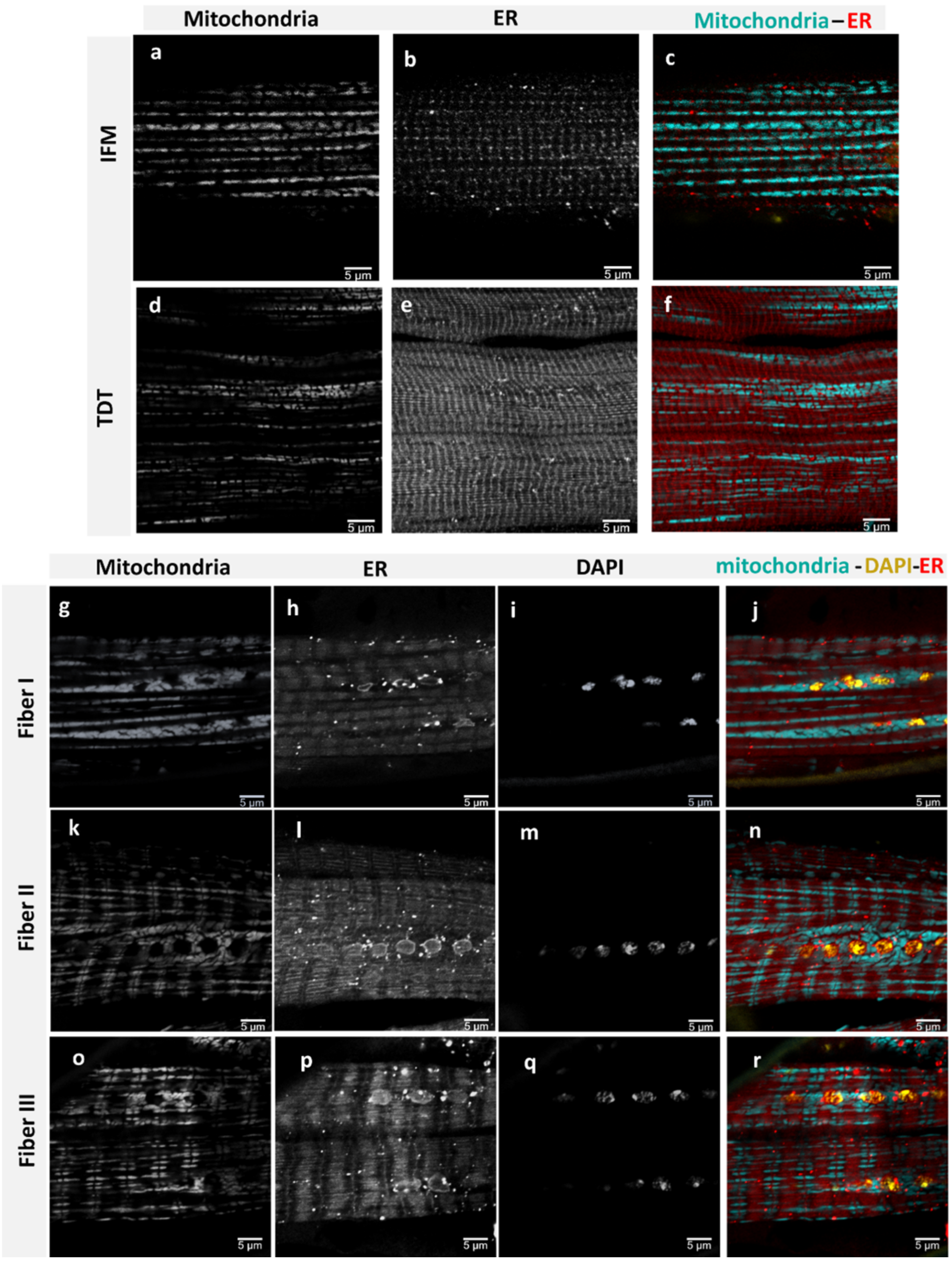

Supplementary Fig. S1. Endoplasmic Reticulum in adult *Drosophila* muscles.

(a, b, c) Wildtype flight muscles (IFMs) showing thin endoplasmic reticulum (ER) (KDEL-rfp) and parallel mitochondria (mito-gfp). (d, e, f) Wildtype jump muscles (TDT) show abundant ER (KDEL-rfp) and parallel mitochondria (mito-gfp). (g-j) Wildtype Leg muscle Fiber I, (k-n) Fiber II, and (o-r) Fiber III stained for mitochondria (mito-gfp), ER (KDEL-rfp), and nuclei (DAPI) (Scale bars: 5 µm).

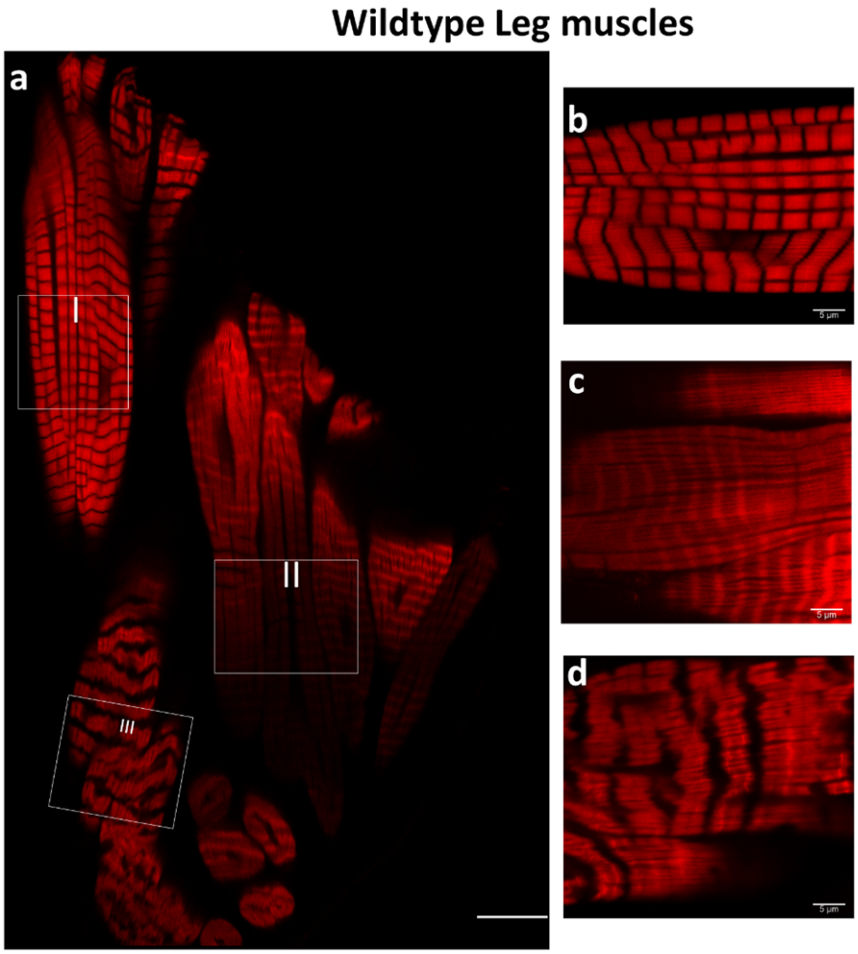

Supplementary Fig. S2. Muscle organization in adult *Drosophila* leg muscles

(a) Wildtype leg muscles (coxa) stained for F-actin (phTRITC) showing three distinct tubular muscle Fiber types (highlighted by squares, scale bar: 20 µm). (b) Tubular muscle fiber of Fiber I. (c) Tubular muscle fiber of Fiber II (d) Tubular muscle fiber of Fiber III (Scale bars: 5 µm).

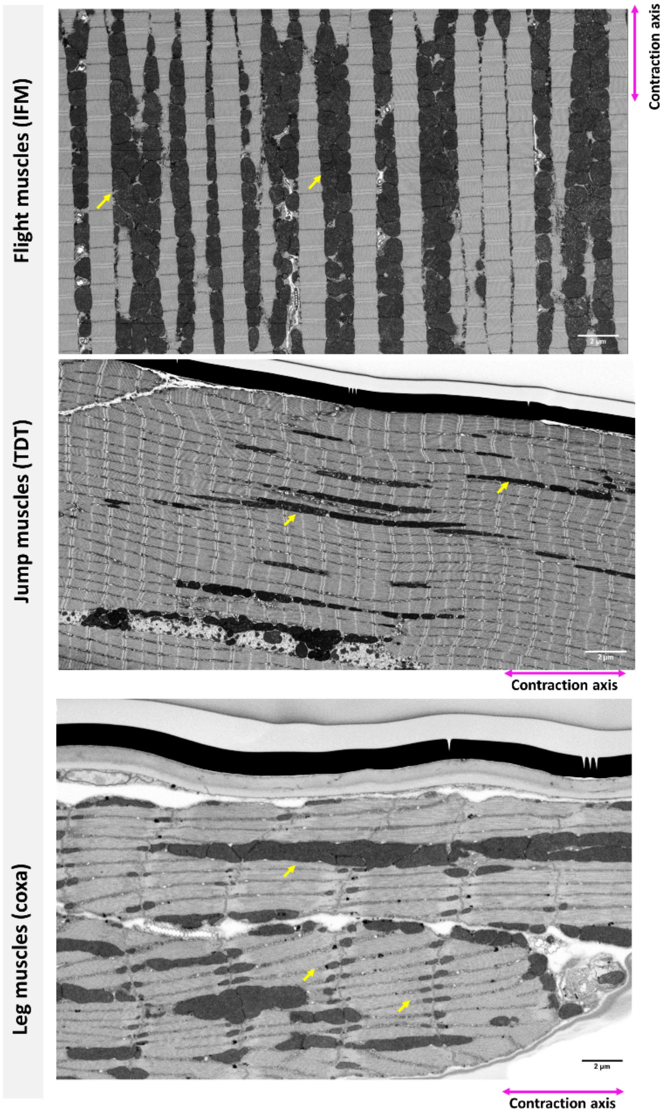

Supplementary Fig. S3. Mitochondrial organization in adult *Drosophila* muscles.

(a, b, c) Electron micrographs showing mitochondrial organization in wildtype (a) flight, (b) jump, and (c) leg muscles. Arrows indicate mitochondria. (Scale bars: 2 µm).

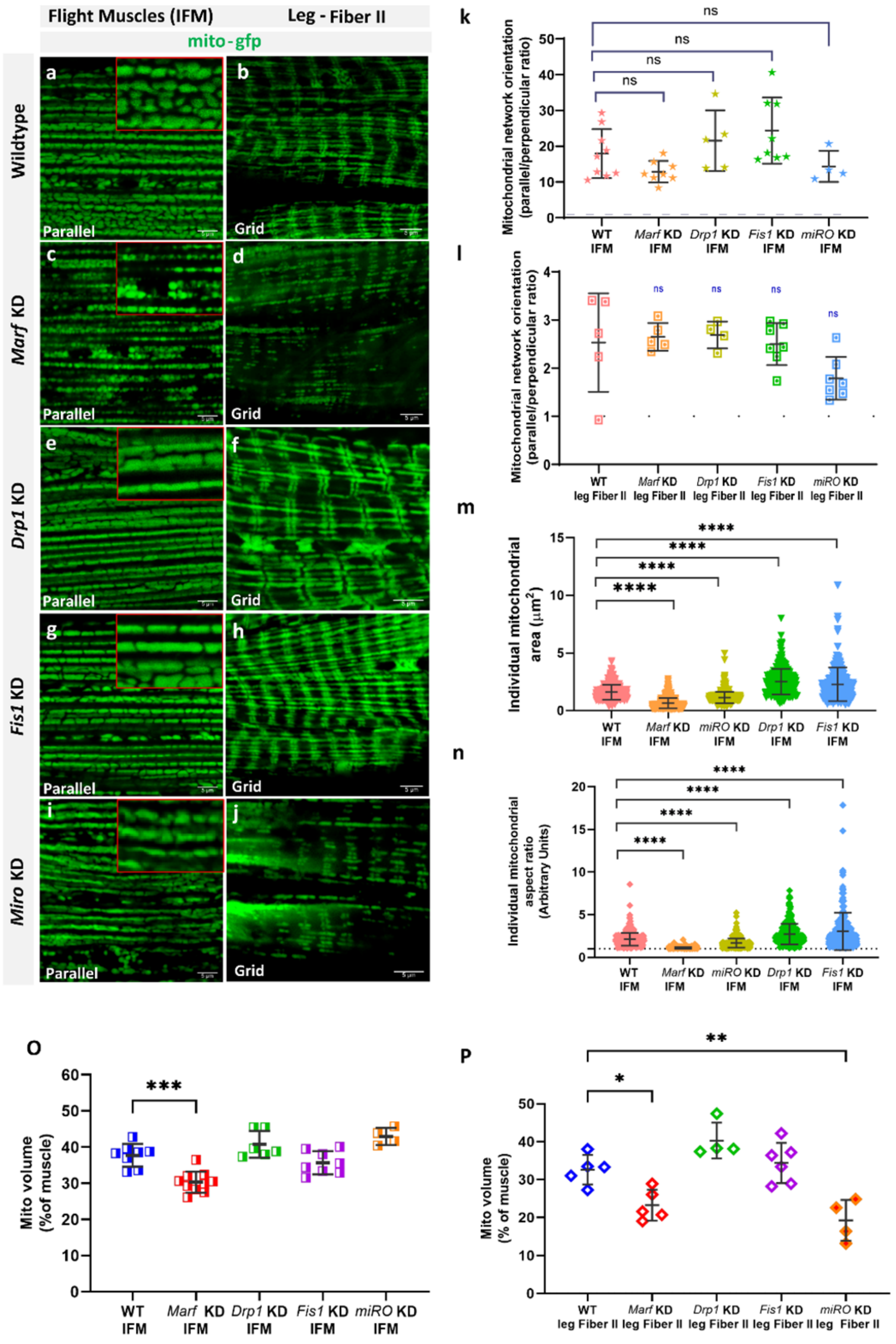

Supplementary Fig. S4. Knockdown of mitochondrial dynamics regulators in *Drosophila* muscles does not affect mitochondrial networks.

(a) Adult wildtype flight muscles (IFM) stained for mitochondria (mito-GFP) reveal tubular mitochondria (inset image) and parallel mitochondrial networks. (b) Wildtype leg muscles showing grid mitochondrial networks (mito-GFP). (c) *Marf* KD in IFM results in circular and small individual mitochondria (inset image) that remain in parallel mitochondrial networks. (d) *Marf* KD in leg muscles shows grid-like mitochondrial networks. (e) *Drp1* KD in flight muscles shows longer mitochondria (inset image), but mitochondrial networks remain parallel. (f) *Drp1* KD leg muscles show grid-like mitochondrial networks. (g) *Fis1* KD in flight muscle shows parallel mitochondrial networks. (h) *Fis1* KD in tubular leg muscles shows grid-like mitochondrial networks. (i) Motility factor, *Miro* KD flight muscles shows abnormal mitochondria morphology (inset image), but retains parallel mitochondrial networks. (j) *Miro* KD in leg muscles showing grid-like mitochondrial networks (Scale bars: 5 µm). (k, l) Quantification of mitochondrial network orientation in (k) IFM and (l) leg muscle fibers. Dotted line represents parallel equal to perpendicular. *mito-gfp;mito-mcherry;Mef2-Gal4* used as Wildtype. (WT-IFM, *n=9 animals*; *Marf* KD-IFM, *n=8 animals*; *Drp1* KD-IFM, *n=5* animals; *Fis1* KD-IFM, *n=8* animals; Miro KD-IFM, *n=4* animals; WT-Leg Fiber II, *n=5* animals; *Marf* KD-Leg Fiber II, *n=5* animals; *Drp1* KD-Leg Fiber II, *n=4* animals; *Fis1* KD-Leg Fiber II, *n=7* animals; *Miro* KD-Leg Fiber II, *n=7* animals). Each point represents value for each animal dataset. (m) Quantification of individual mitochondrial area and (n) quantification of individual mitochondrial aspect ratio (major axis/minor axis) (WT-IFM, *n=393; Marf* KD-IFM, *n=1091*; *Miro* KD-IFM, *n=509; Drp1* KD-IFM, *n=329*; *Fis1* KD-IFM, *n=356*). Each point represents value for individual mitochondria. (o, p) Quantification of mitochondrial volume as a percent of total muscle volume in (o) IFM and (p) leg Fiber II. *UAS-mito-gfp;UAS-mito-OMM-mcherry;Dmef2-Gal4* used as wildtype. (WT-IFM, *n=8* animals; *Marf* KD-IFM, *n=9* animals; *Drp1* KD-IFM, *n=6* animals; *Fis1* KD-IFM, *n=8* animals; *Miro* KD-IFM, *n=4* animals; WT-Fiber II, *n=5* animals ; *Marf* KD-Fiber II, *n=5* animals; *Drp1* KD-Fiber II, *n=4* animals; *Fis1* KD-Fiber II, *n=6* animals; *Miro* KD-Fiber II, *n=4* animals). Bars represent mean ± SD. Significance determined as $p < 0.05$ from one way ANOVA with Tukey's (*, $p \le 0.05$; **, $p \le 0.01$; ***, $p \le 0.001$; ****, $p \le 0.0001$; ns, non-significant).

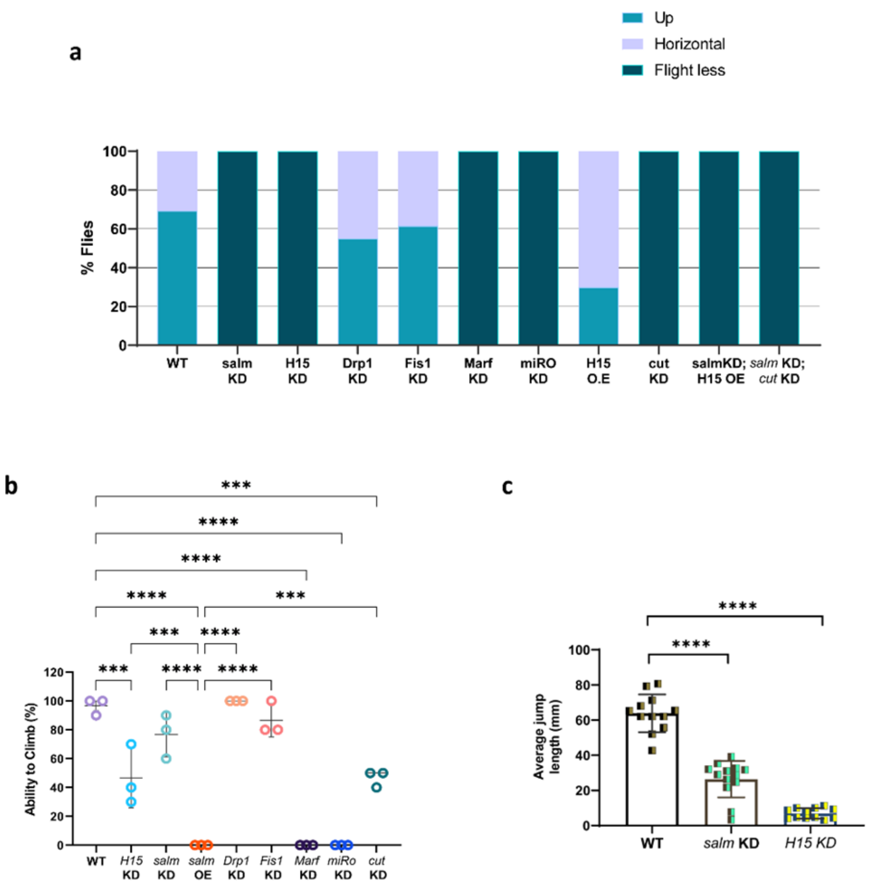

Supplementary Fig. S5. Flight, climbing, and jump ability. (a) Flight assay. *mito-gfp;Dmef2-Gal4* used as wildtype (WT, *n=13*; *salm* KD, *n=26*; *H15* KD, *n=22*; *Marf* KD, *n=25*; *Drp1* KD, *n=20*; *Fis1* KD, *n=13*; *Miro* KD, *n=13*; *H15* OE, *n=10; cut* KD, *n=20*, *salm* KD;*H15* OE, *n=13*; *salm* KD; *cut* KD, *n=24*). (f) Climbing assay. Each point represents one group (*n=10* for each group). (g) Average jump length (WT, *n=12*; *salm* KD, *n=13*; *H15* KD, *n=12*). Each point represents individual fly. Bars represent mean ± SD. Significance determined as p < 0.05 from one way ANOVA with Tukey's (*, p ≤ 0.05; **,p ≤ 0.01; ***,p ≤ 0.001; ****, p ≤ 0.0001; ns, non-significant).

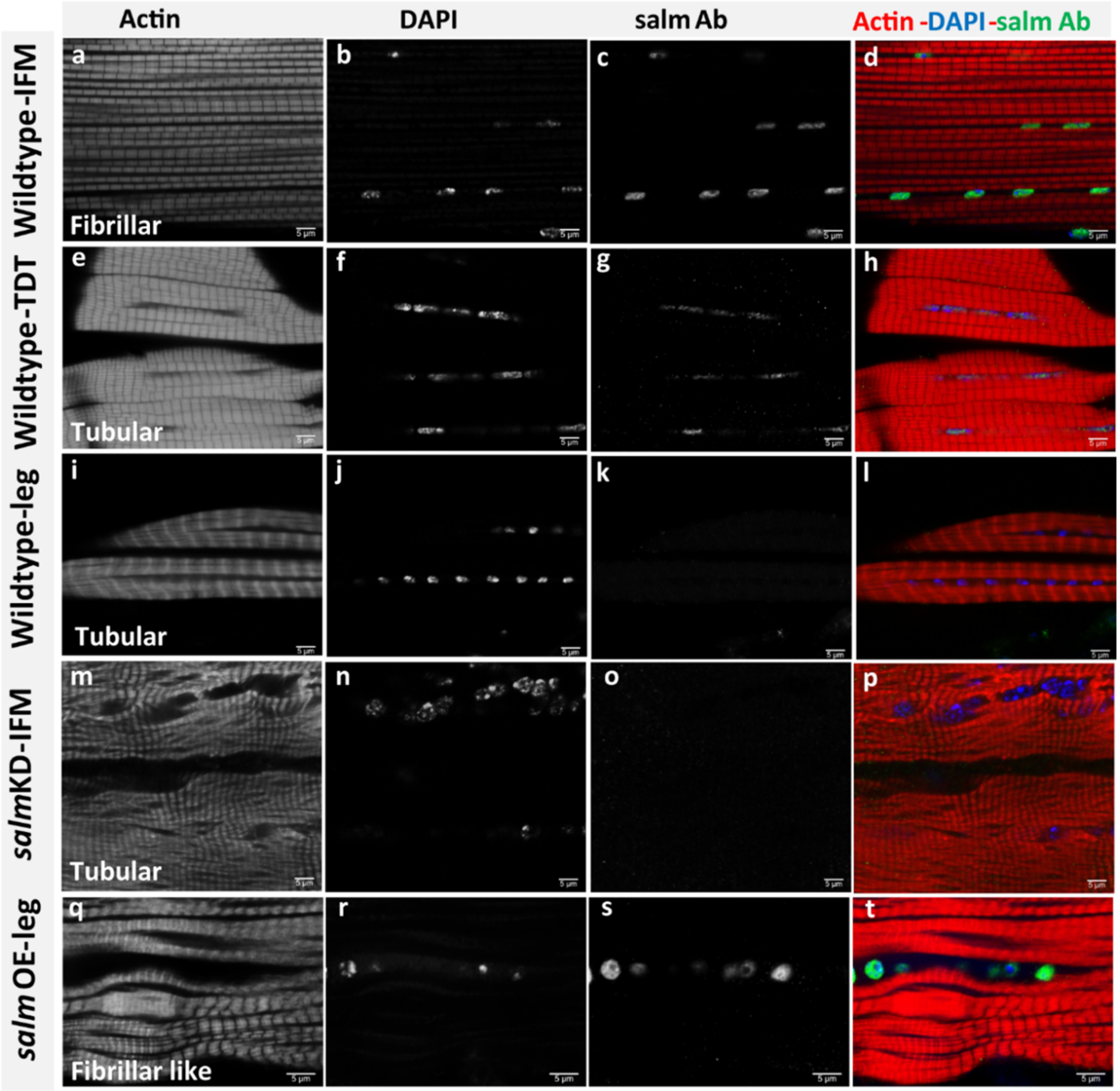

Supplementary Fig. S6. salm expression in *Drosophila* muscles.

(a-d) Wildtype flight muscles (IFM), (e-h) jump muscles, and (i-l) leg muscles stained for F-actin (phTRITC), nuclei (DAPI), and Salm antibody. (m-p) *salm* KD IFM showing decreased expression of Salm in nuclei (DAPI). (q-t) Leg muscles with *salm* OE stained for F-actin (phTRITC), nuclei (DAPI), and Salm antibody showing increased Salm expression in the nuclei (Scale Bars: 5 µm for all).

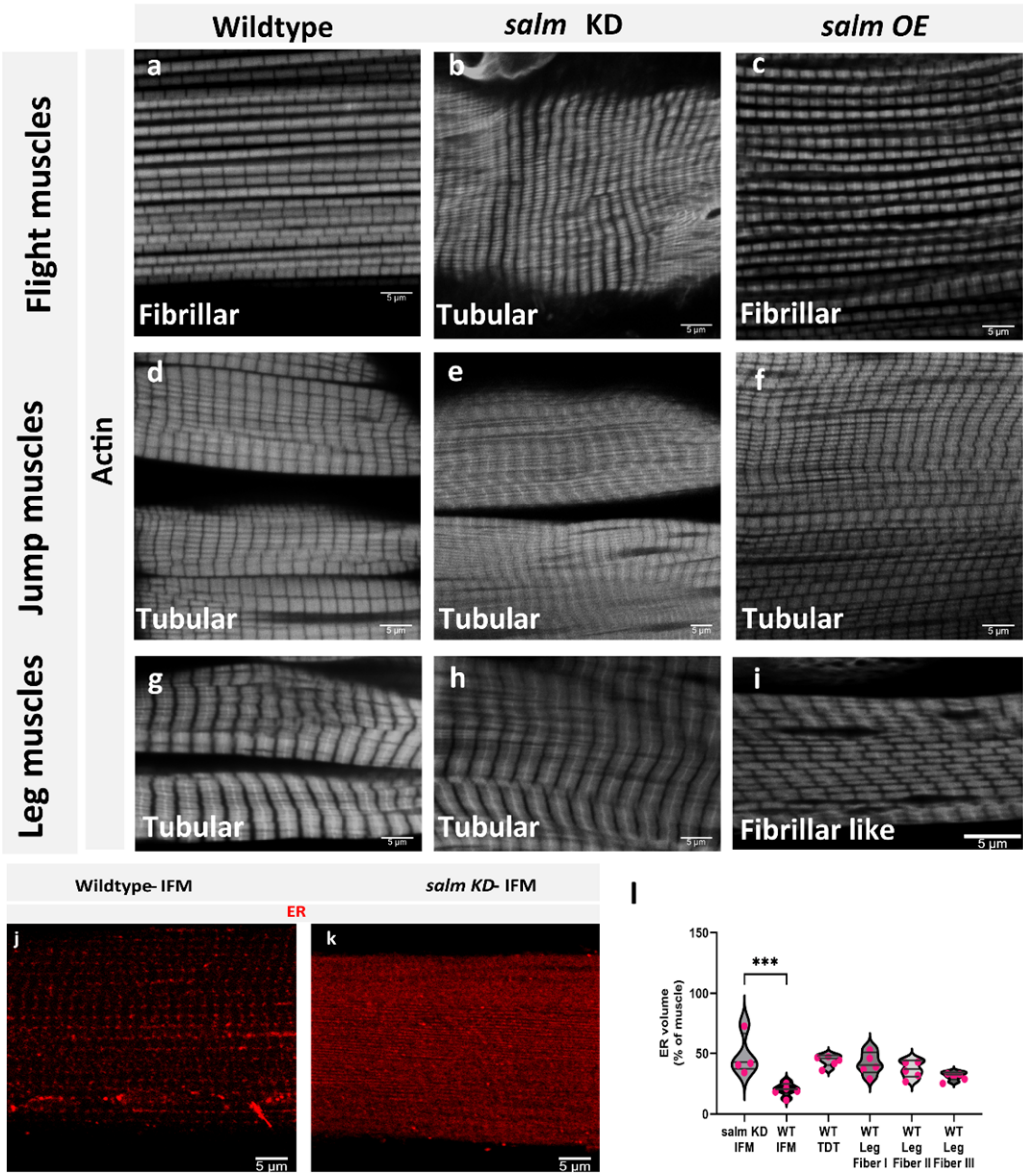

Supplementary Fig. S7. *salm* overexpression results in conversion of contractile type in leg muscles.

(a, b, c) Adult flight muscles (IFM) stained for F-actin (phTRITC) showing change in fiber type from (a) wildtype fibrillar to tubular fibers in (b) *salm* KD, but not in (c) *salm* OE. (d, e, f) Tubular jump muscles do not show alteration with either *salm* KD or *salm* OE. (g, h, i) Muscle contractile fiber type of wildtype leg muscles undergo conversion upon *salm* OE, but not *salm* KD. (j, k) Flight muscles stained for endoplamic reticulum (KDEL-rfp) showing increased ER content in (k) salm KD tubular IFMs compared to (j) wildtype fibrillar flight muscles (Scale bars: 5 µm). (l) Quantification of ER volume as a percent of muscle volume (*salm* KD-IFM, *n*=4 animals*;* WT-IFM, *n=5* animals; Jump muscles (TDT), *n=5* animals; WT Leg Fiber I, *n=5* animals; WT Leg Fiber II, *n=5* animals; WT Leg Fiber III, *n=5* animals). Each point represents value for each animal dataset. Bars represent mean ± SD. Significance determined as $p < 0.05$ from one way ANOVA with Tukey's (*, $p \le 0.05$; **, $p \le 0.01$; ***, $p \le 0.001$; ****, $p \le 0.0001$; ns, non-significant).

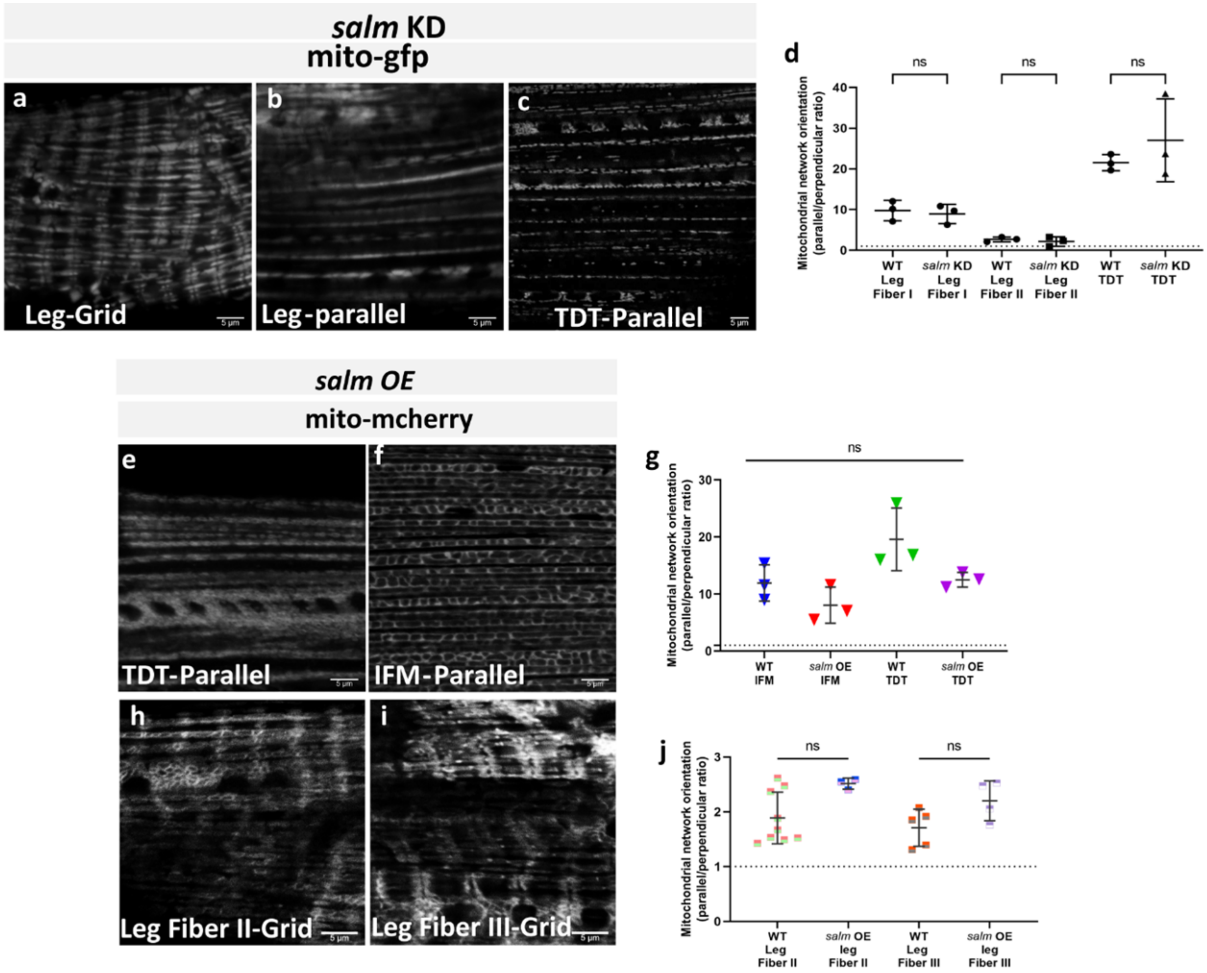

Supplementary Fig. S8. Effect of s*alm* knockdown and overexpression on mitochondrial networks in muscles.

(a, b, c) Mitochondrial networks (mito-gfp) in *salm* KD (a, b) leg muscles and (c) jump (TDT) muscles (Scale bars: 5 µm). (d) Quantification of mitochondrial network orientation. Dotted line represents parallel equal to perpendicular. *mito-gfp;mito-mcherry;Mef2-Gal4* used as Wildtype (*n=3 animals* for all groups). (e, f) Mitochondrial networks (mito-mcherry) in (e) jump and (f) flight muscles after *salm* OE (Scale bars: 5 µm for all). (g) Quantification of mitochondrial network orientation. Dotted line represents parallel equal to perpendicular. *mito-gfp;mito-mcherry;Mef2-Gal4* used as Wildtype (*n=3* animals for all groups). (h, i) Mitochondrial networks (mito-mcherry) in (h) Fiber II and (i) Fiber III of leg muscles after *salm* OE. (j) Quantification of mitochondrial network orientation in leg muscle fibers. Dotted line represents parallel equal to perpendicular. *mito-gfp;mito-mcherry;Mef2-Gal4* used as Wildtype (WT-Leg Fiber II, *n=9* animals; *salm* OE-Leg Fiber II, *n=3* animals; WT-Leg Fiber III, *n=5* animals; *salm* OE-Leg Fiber III, *n=4* animals). Each point represents value for each animal dataset. Bars represent mean ± SD. Significance determined as $p < 0.05$ from one way ANOVA with Tukey's (*, $p \le 0.05$; **,$p \le 0.01$; ***,$p \le 0.001$; ****, $p \le 0.0001$; ns, non-significant).

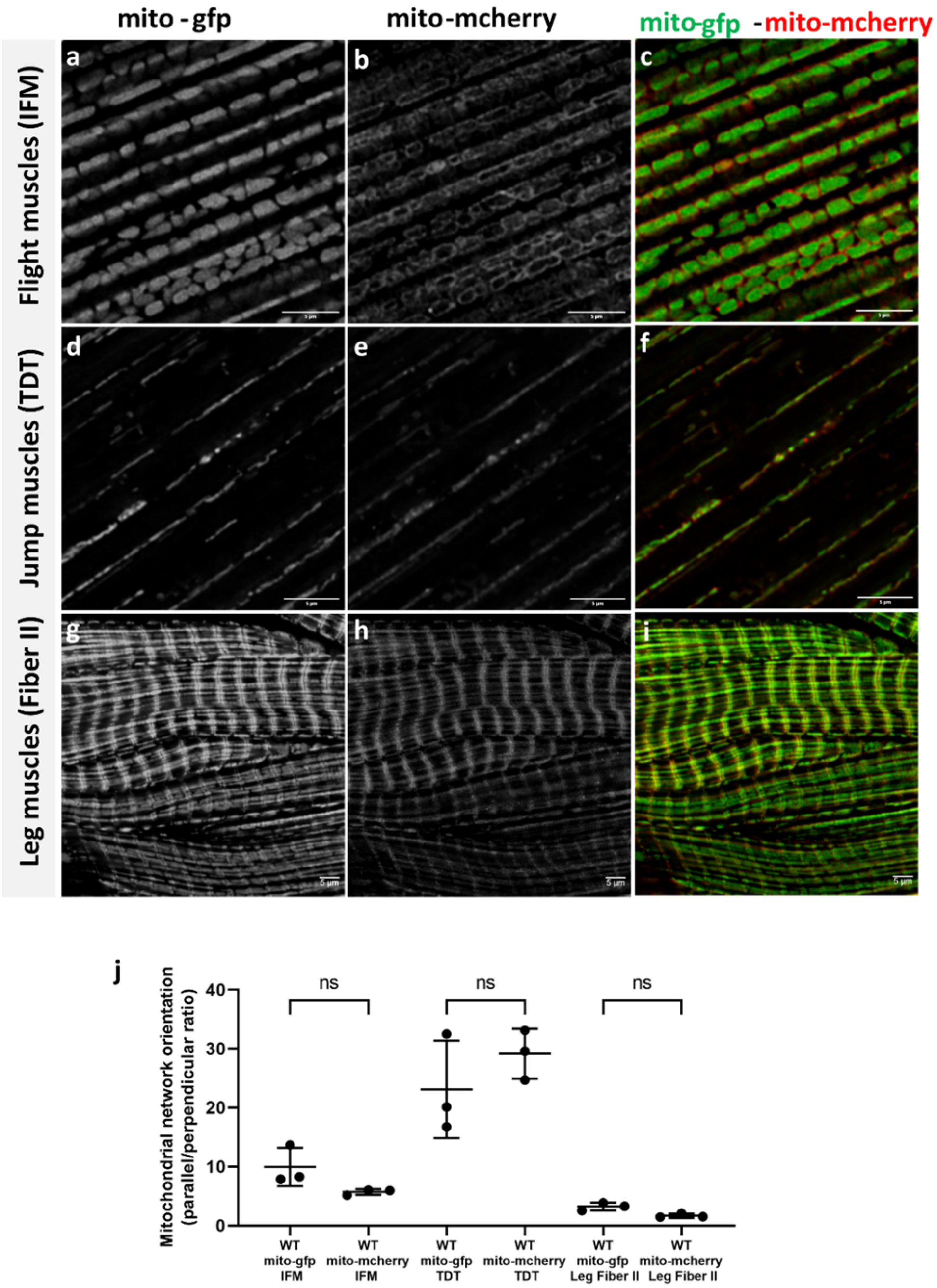

Supplementary Fig. S9. Mitochondrial network organization in adult *Drosophila* muscles

(a, b, c) Wildtype IFM with parallel mitochondria (mito-gfp and mito-mcherry). (d, e, f) Wildtype jump muscles showing parallel mitochondria. (g, h, i) Wildtype leg muscles showing grid-like mitochondria (mito-gfp and mito-mcherry) in Fiber II (Scale Bars: 5 µm). (j) Quantification of mitochondrial network orientation visualized by mito-gfp and mito-mcherry (IFM, n=3 animals; TDT, n=3 animals; Leg fiber II, n=3 animals). Dotted line represents parallel equal to perpendicular. *mito-gfp;mito-mcherry;Mef2-Gal4* used as Wildtype (*n=3* for all groups) Bars represent mean ± SD. Significance determined as $p < 0.05$ from one way ANOVA with Tukey's (ns, non-significant).

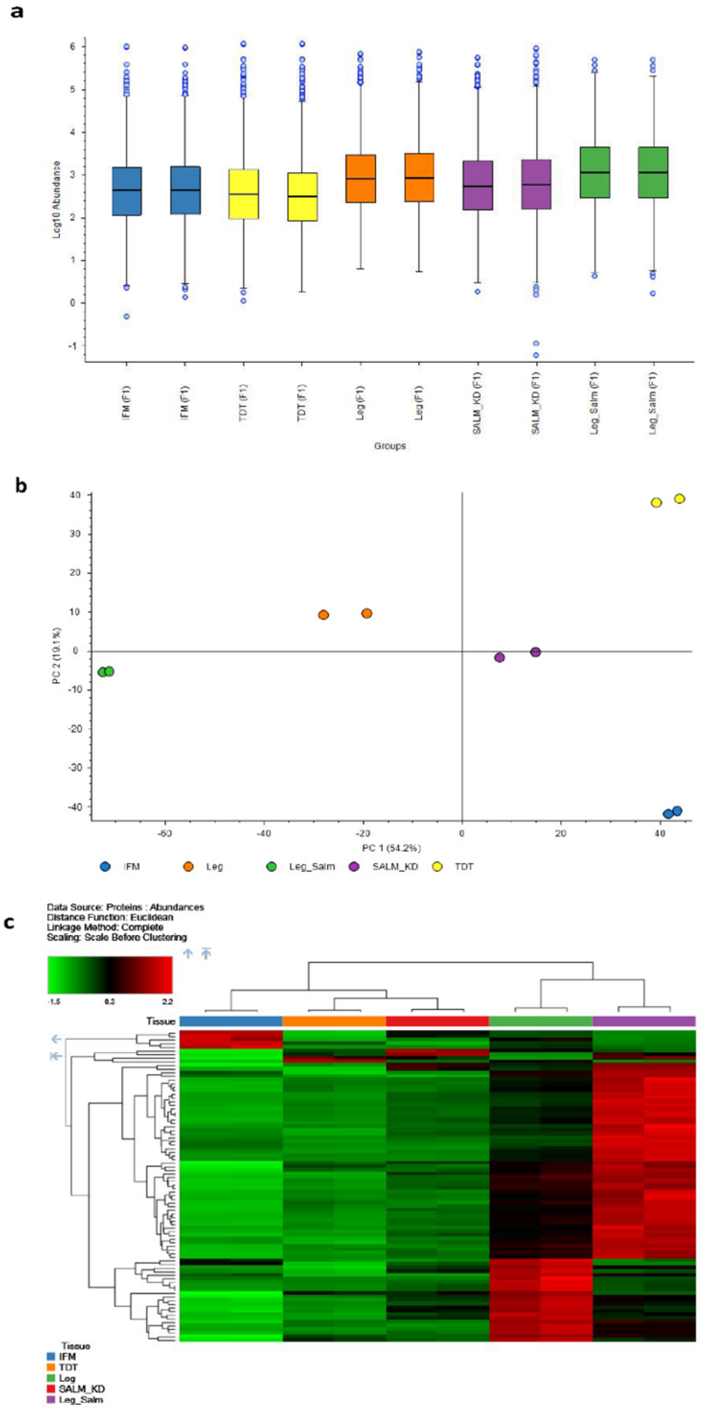

Supplementary Fig. S10. Principal component analysis (PCA) and heatmap of proteome data.

(a) Comparison of protein abundance between muscle samples. The box-and-whisker plot shows the abundance of the intensity values for each individual muscle group. (b) Principal component analysis of the proteome data in a 2D graph of PC1 and PC2. (c) Heatmap of protein abundance pattern in differentially expressed proteins showing minimum value, maximum value, and mean. Error bars indicate standard deviation.

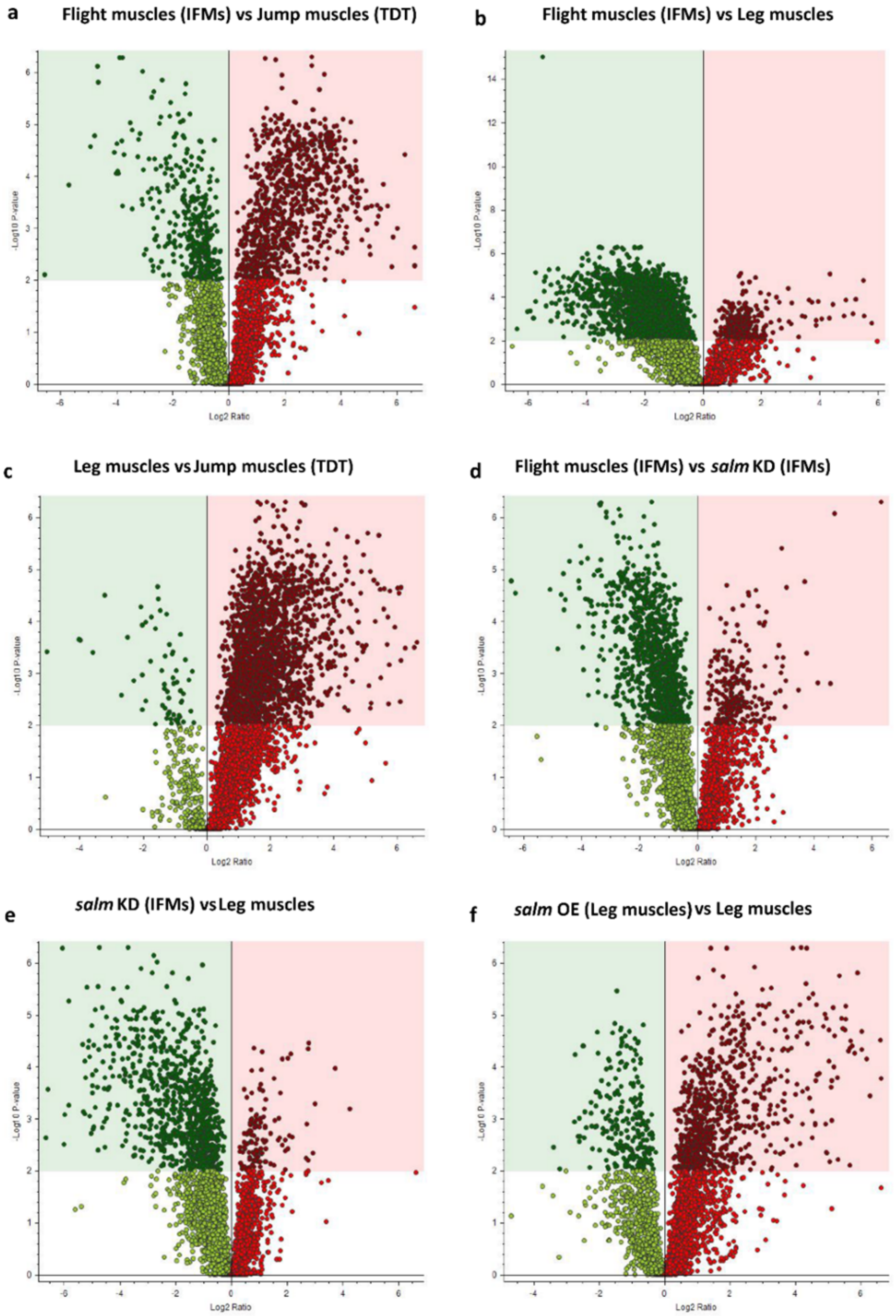

Supplementary Fig. S11. Volcano plots showing differential protein expression between muscle types.

(a) Flight muscles (IFM) vs. jump muscles (TDT). (b) Flight muscles vs. leg muscles. (c) Leg muscles vs jump muscles. (d) Wildtype flight muscles vs. *salm* KD flight muscles. (e) *salm* KD flight muscles vs. wildtype leg muscles. (f) *salm* OE leg muscles vs. wildtype leg muscles. Green dots represent proteins with significantly lower abundances, while the red dots show proteins with higher levels of expression. X-axis, log 2 fold

change (FC) differences in the gene expression and Y-axis, negative log 10 of the p values. Significance determined as p = 0.01 from one way ANOVA with Tukey's.

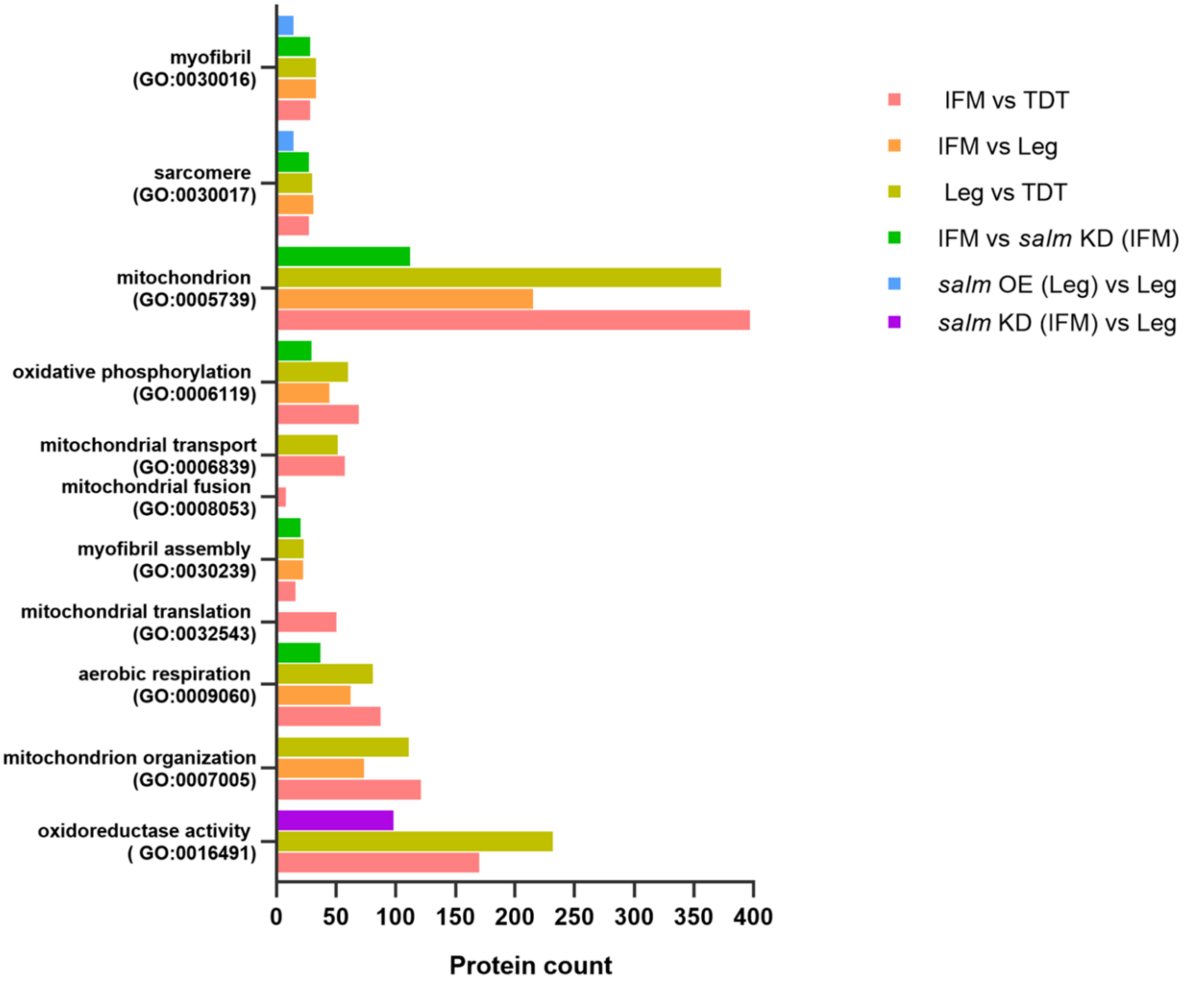

Supplementary Fig. S12. Gene enrichment analysis of differentially expressed proteins among the five different muscle types using g:profiler.

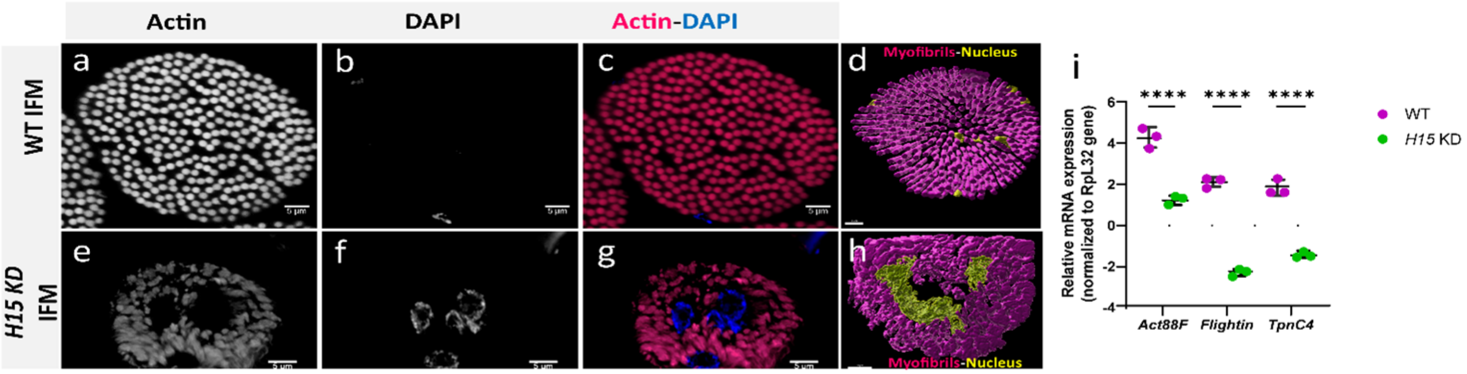

Supplementary Fig. S13. ***H15*** **Knockdown results in conversion of contractile type to tubular from of fibrillar flight muscles.**

(a, b, c) Adult flight muscles (IFM) stained for F-actin (phTRITC) showing wildtype fibrillar flight muscles. (d) 3D Rendered fibrillar flight muscles. (e, f, g) *H15* Knockdown changes in fiber type from wildtype fibrillar to tubular fibers. (h) 3D Rendered H15 KD- flight muscles.(i) Quantification of transcript levels of sarcomeric genes *Act88F*, *Flightin* and *Tpnc4*. Each point represents value for each dataset. Bars represent mean ± SD. Significance determined as $p < 0.05$ from one way ANOVA with Tukey's (*, $p \leq 0.05$; **,$p \leq 0.01$; ***,$p \leq 0.001$; ****, $p \leq 0.0001$; ns, non-significant).

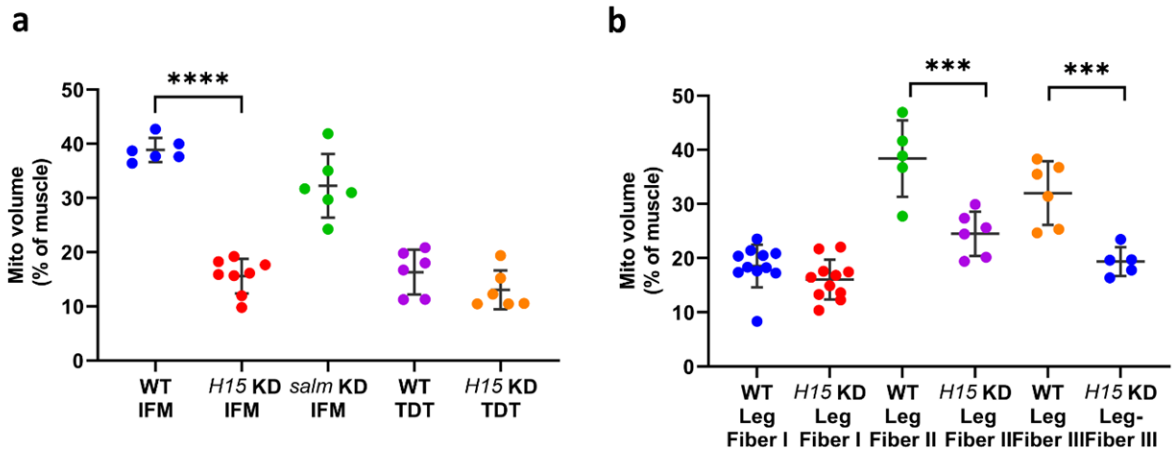

Supplementary Fig. S14. Effect of *H15* knock down on mitochondrial volume

(a, b) Quantification of mitochondrial volume as a percent of total muscle volume in (a) IFM and (b) leg muscle fibers. *UAS-mito-gfp;UAS-mito-OMM-mcherry;Dmef2-Gal4* used as wildtype. (WT-IFM, *n=6* animals; *H15* KD-IFM, *n=7* animals; *salm* KD-IFM, *n=6* animals; WT-TDT, *n=6* animals; *H15* KD-TDT, *n=5* animals; WT-Leg Fiber I, *n=11* animals; *H15* KD-Leg Fiber I, *n=11* animals; WT-Leg Fiber II, *n=5* animals; *H15* KD-Leg Fiber II, *n=6* animals; WT-Leg Fiber III, *n=6* animals; *H15* KD-Leg Fiber III, *n=5* animals). Bars represent mean ± SD. Significance determined as $p < 0.05$ from one way ANOVA with Tukey's (*, $p \le 0.05$; **,$p \le 0.01$; ***,$p \le 0.001$; ****, $p \le 0.0001$; ns, non-significant).

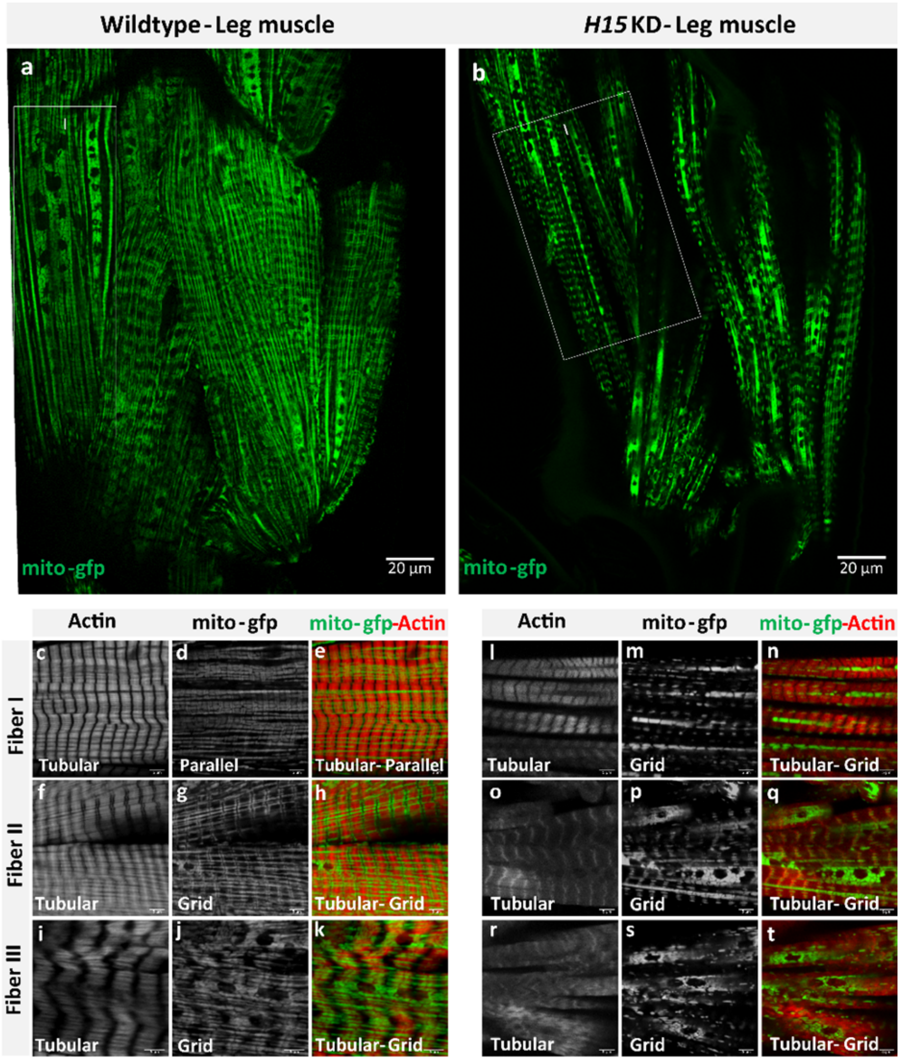

Supplementary Fig. S15. *H15* regulates conversion of mitochondrial networks, but not contractile type in *Drosophila* leg muscles.

(a) Adult wildtype leg coxa muscles showing parallel mitochondrial networks (mito-gfp) in Fiber I (marked within rectangle) and grid-like networks in other regions. (b) *H15* KD leg muscles showing a uniform grid-like mitochondrial networks, even in fiber I (marked by rectangle) (Scale bar: 20 µm). (c, d, e) Wildtype leg muscle Fiber I showing parallel mitochondrial networks (mito-gfp) and tubular muscle fiber (phTRITC). (f, g, h) Wildtype leg muscle Fiber II and (i, j, k) Fiber III showing grid-like mitochondrial networks and tubular myofibrils. (l, m, n) *H15* KD leg muscles Fiber I showing grid-like mitochondrial networks, unlike wildtype Fiber I, but myofibrils remain tubular. (o, p, q) H15 KD leg muscle Fiber II and (r, s, t) Fiber III showing grid-like mitochondrial networks and tubular muscle fibers as in wildtype (Scale Bars: 5 µm).

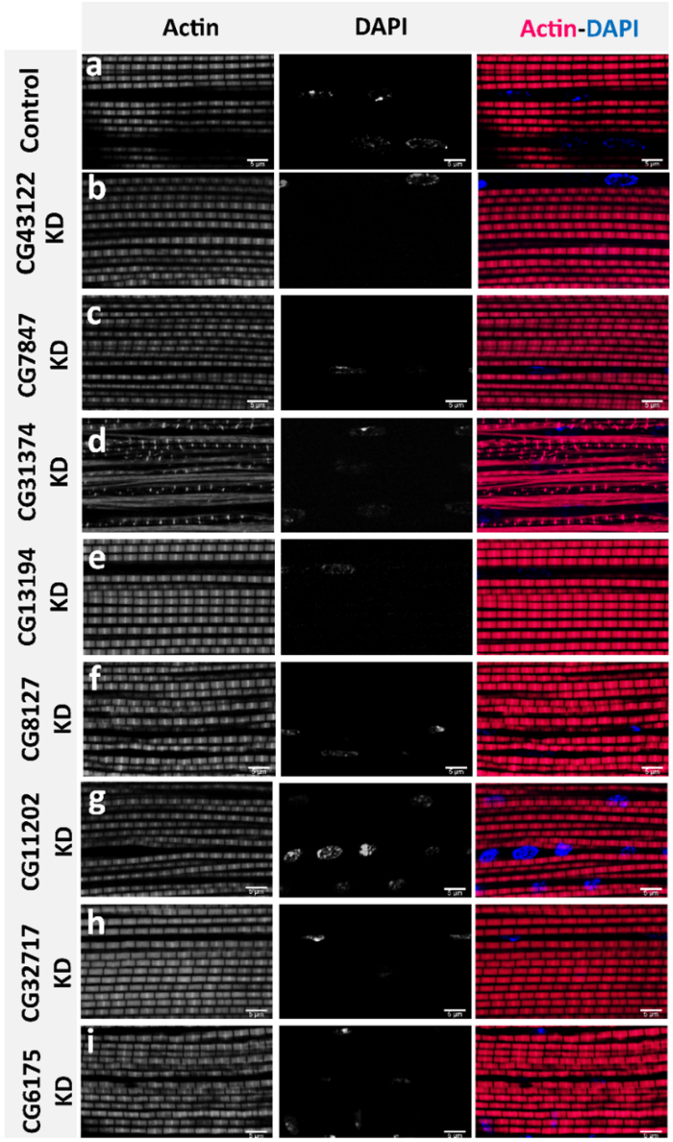

Supplementary Fig. S16 Knockdown of H15 RNAi line (V28415) off-targets does not result in fiber type change in flight muscles.

Adult flight muscles with myofibrils stained for ph-TRITC and nuclei (DAPI) in (a) Wildtype, (b) CG43122 KD, (c) CG7847 KD, (d) CG31374 KD, (e) CG13194 KD, (f) CG8127 KD, (g) CG11202 KD, (h) CG32717 KD, and (i) CG6175 KD flies.

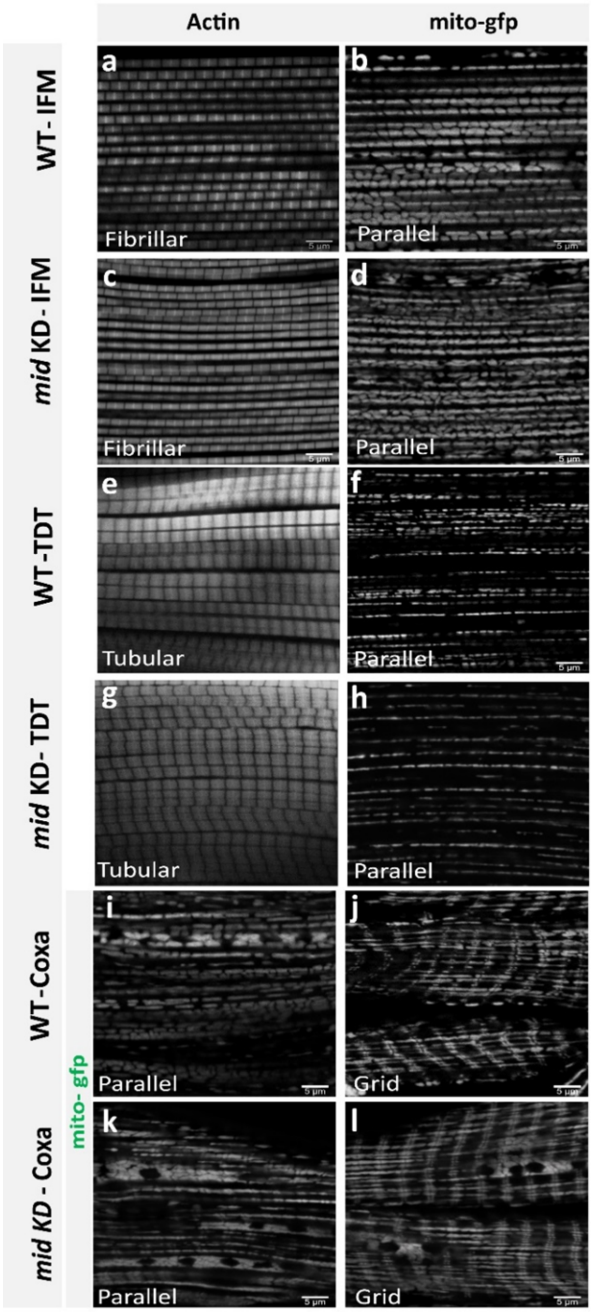

Supplementary Fig. S17. Effect of *mid* knockdown on mitochondrial networks in muscles.

(a, b) Mitochondrial networks (mito-gfp) in WT-IFM. (c, d) Parallel mitochondrial networks (mito-gfp) in *mid* KD-IFM. (e, f) WT jump (TDT) muscles showing parallel mitochondrial networks. (g, h) *mid* KD-jump (TDT) muscles showing parallel mitochondrial networks. (I, j) WT- Coxa leg Fiber I and Fiber II show parallel and grid-like mitochondrial networks respectively. (k, l) *mid* KD -Coxa leg Fiber I and Fiber II show parallel and grid-like mitochondrial networks respectively similar to WT- Coxa leg Fiber I and Fiber II (Scale bars: 5 µm for all).

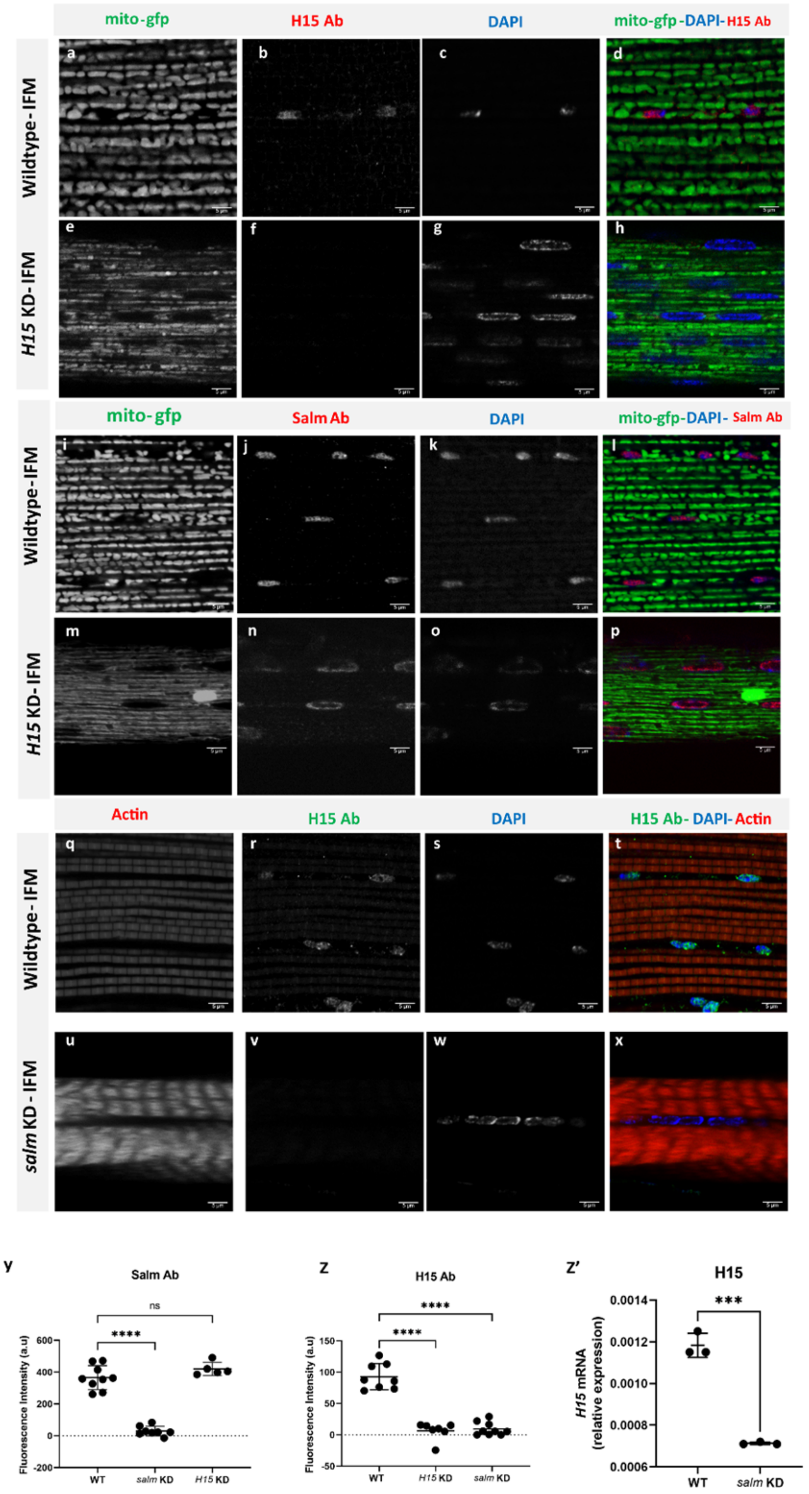

Supplementary Fig. S18. ***H15*** **is downstream of** ***salm*** **in the fiber type specification pathway.**

(a-d) Wildtype IFM stained for mitochondria (mito-gfp), H15 antibody, and nuclei (DAPI) showing H15 expression in the nuclei. (e-h) *H15* KD IFM showing decreased expression of H15. (i-l) Wildtype IFM stained for mitochondria (mito-gfp), salm antibody, and nuclei (DAPI) showing Salm expression in the nuclei. (m-p) In *H15* KD IFM, Salm expression is unaffected. (q-t) Wildtype IFM stained for mitochondria (mito-gfp),

H15 antibody, and nuclei (DAPI). (u-x) *salm* KD IFM showing decreased expression of H15 (Scale Bars: 5 µm for all). (y, z) Quantification of fluorescence intensity of (y) salm antibody staining (WT-IFM, *n=8*; *salm* KD-IFM, *n=8*; *H15* KD-IFM, *n=5)* and (z) H15 antibody staining (WT-IFM, *n=8*; *H15* KD-IFM, *n=7; salm* KD-IFM, *n=9)*. (z') Quantification of transcript levels of *H15.* Each point represents value for each dataset. Bars represent mean ± SD. Significance determined as $p < 0.05$ from one way ANOVA with Tukey's (*, $p \le 0.05$; **,$p \le 0.01$; ***,$p \le 0.001$; ****, $p \le 0.0001$; ns, non-significant).

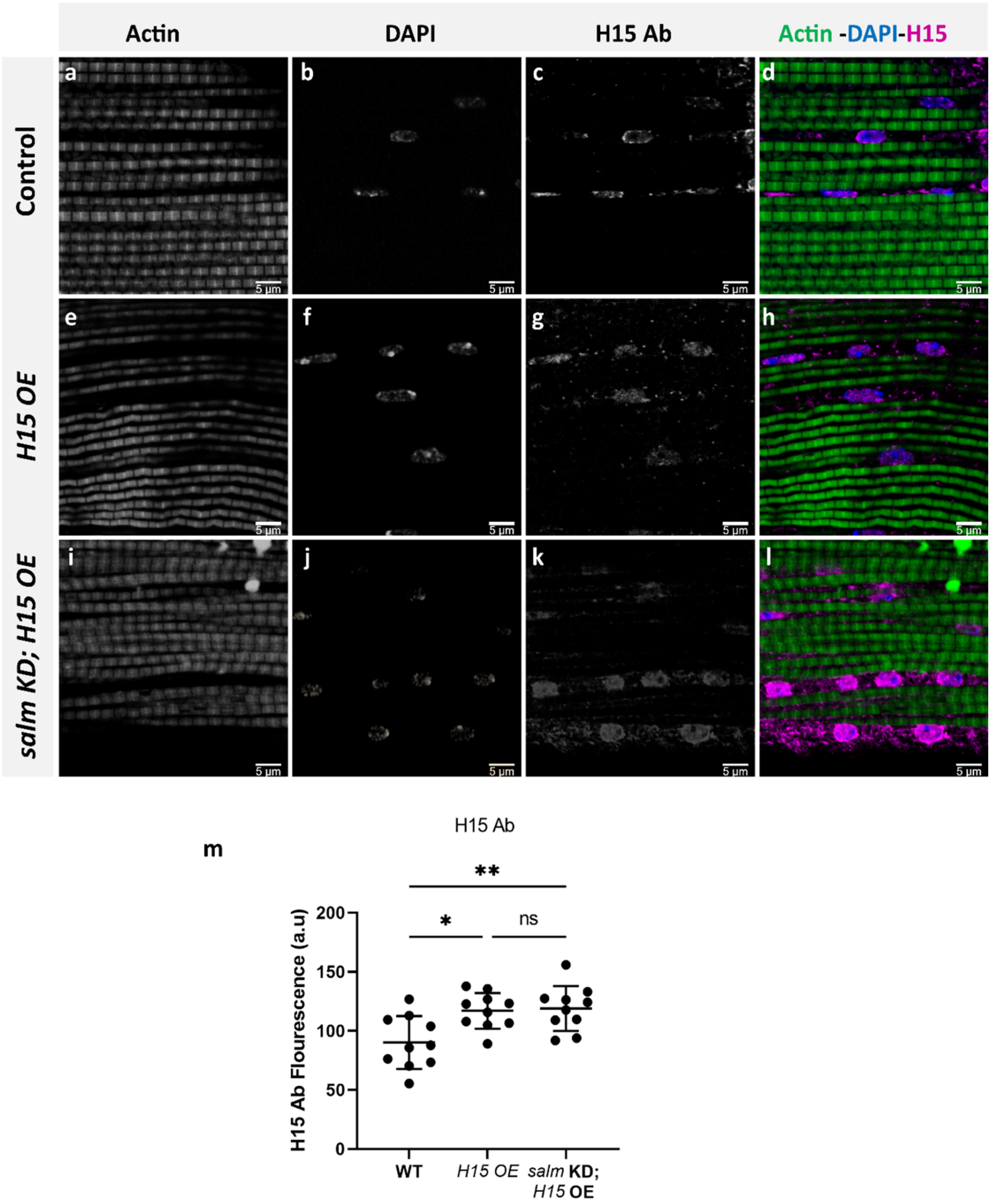

Supplementary Fig. S19. *H15* overexpression in *salm* knock down background

(a-d) Wildtype fibrillar flight muscles (IFMs) stained for F-actin (phTRITC), nuclei (DAPI), and H15 antibody showing H15 expression in the nuclei. (e-h) *H15* OE IFM showing increased expression of H15. (i-l) *H15* OE; *salm* KD IFM showing overexpression of H15 (Scale Bars: 5 µm). (m) Quantification of fluorescence

intensity of H15 antibody staining (WT, n=10 ; H15 OE, n=10 and salm KD; H15 OE, n=10) . Each point represents value for each dataset. Bars represent mean ± SD. Significance determined as $p < 0.05$ from one way ANOVA with Tukey's (*, $p \leq 0.05$; **,$p \leq 0.01$; ***,$p \leq 0.001$; ****, $p \leq 0.0001$; ns, non-significant).

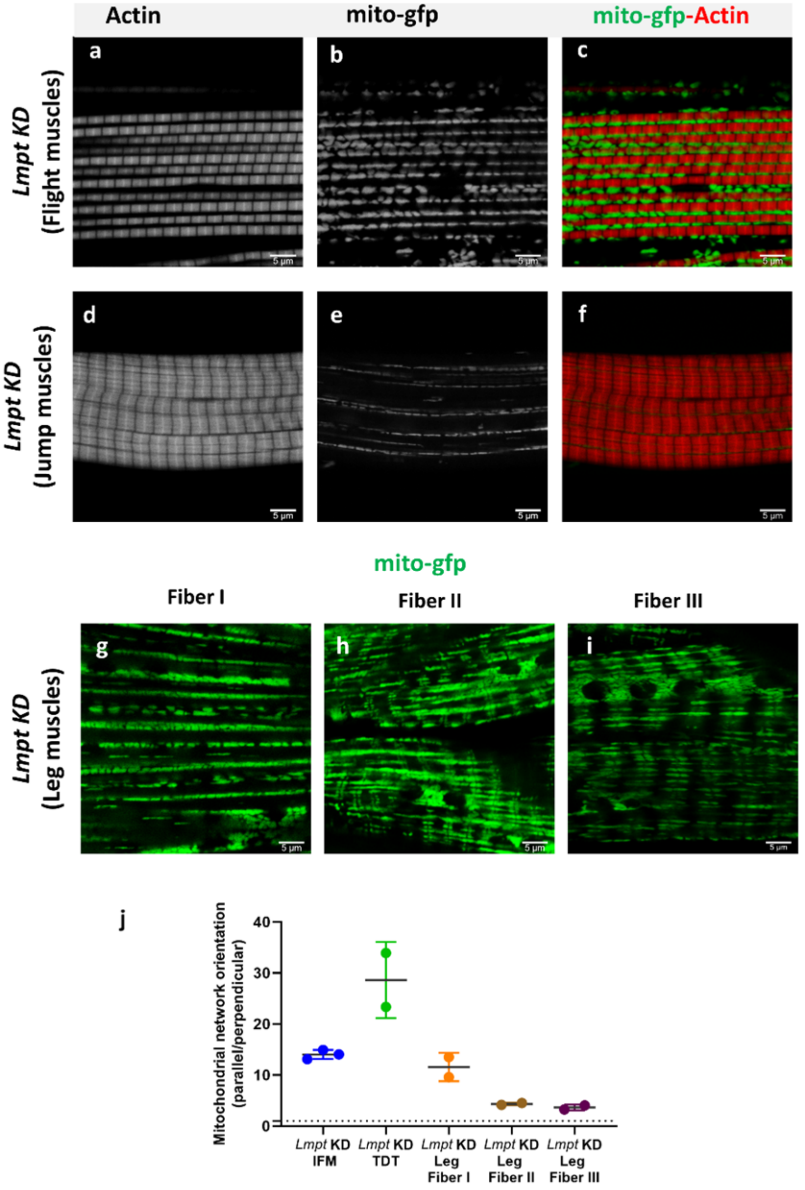

Supplementary Fig. S20. ***lmpt*** **knock down in muscles does not affect mitochondrial networks and contractile type in** ***Drosophila*** **muscles.**

(a, b, c) Adult flight muscles with *lmpt* KD showing fibrillar myofibrils (ph-TRITC) and parallel mitochondrial networks (mito-gfp). (d, e, f) *lmpt* KD jump muscles showing tubular contractile type and grid-like mitochondrial network. (g) Leg muscle Fiber I with *lmpt* KD retains parallel mitochondrial networks (mito-gfp). (h) *lmpt* KD leg muscle Fiber II and (i) *lmpt* KD Fiber III showing grid-like mitochondrial networks (Scale

Bars: 5 µm). (j) Quantification of mitochondrial network orientation. Dotted line represents parallel equal to perpendicular (*Lmpt* KD IFM, n= 3 animals; *Lmpt* KD TDT, n=2 animals; *Lmpt* KD Leg fiber I, n=2 animals; *Lmpt* KD Leg fiber II, n=2 animals; Leg fiber III, n=2 animals). Each point represents value for each animal dataset. Bars represent mean ± SD. Significance determined as p < 0.05 from one way ANOVA with Tukey's (*, p ≤ 0.05; **,p ≤ 0.01; ***,p ≤ 0.001; ****, p ≤ 0.0001; ns, non-significant).

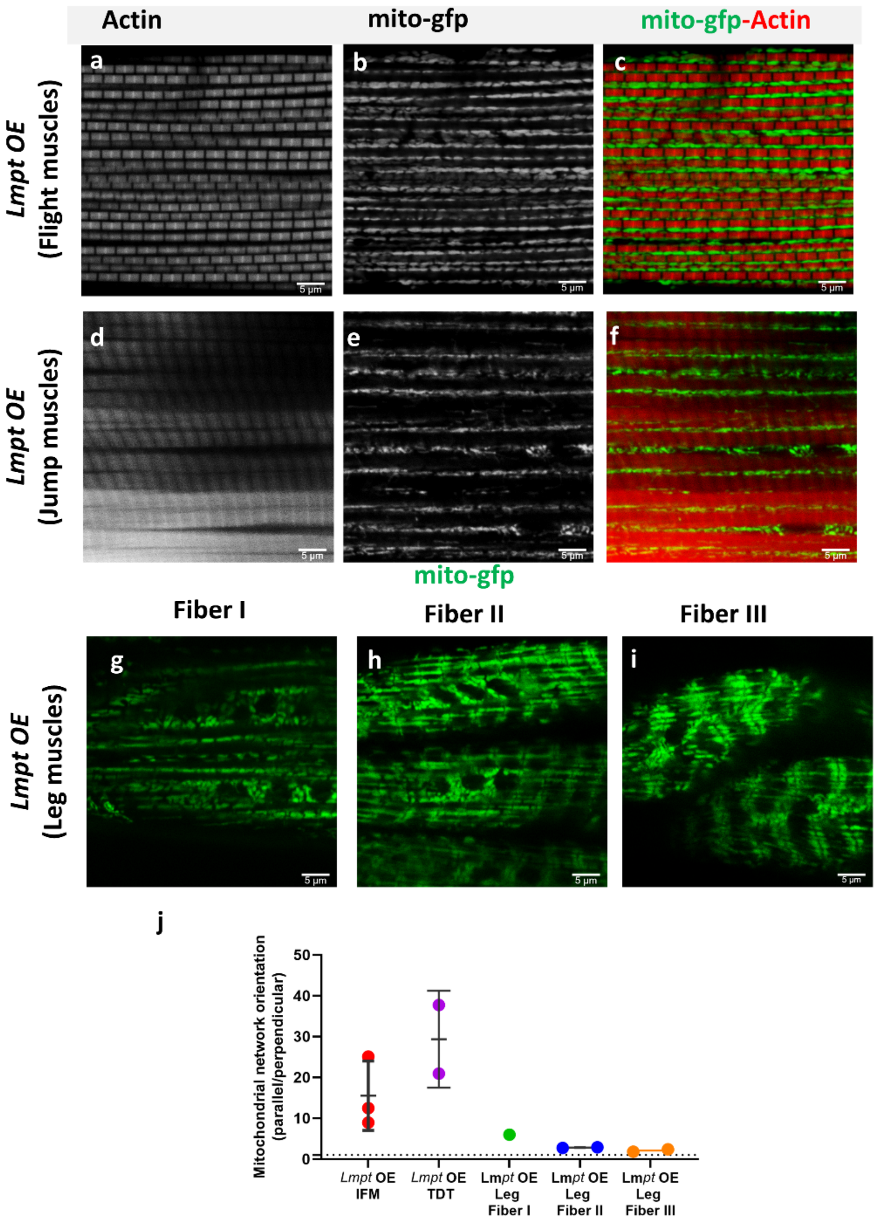

Supplementary Fig. 21. ***lmpt*** **overexpression in muscles does not affect mitochondrial networks and contractile type in** ***Drosophila*** **muscles.**

(a, b, c) Adult flight muscles with *lmpt* OE showing fibrillar myofibrils (ph-TRITC) and parallel mitochondrial networks (mito-gfp). (d, e, f) *lmpt* OE jump muscles showing tubular contractile type and grid-like mitochondrial network. (g) Leg muscle Fiber I with *lmpt* OE retains parallel mitochondrial networks (mito-gfp). (h) *lmpt* OE leg muscle Fiber II and (i) *lmpt* OE Fiber III showing grid-like mitochondrial networks (Scale Bars: 5 µm). (j) Quantification of mitochondrial network orientation. Dotted line represents parallel

equal to perpendicular(*Lmpt* OE IFM, n= 3 animals; Lmpt OE TDT, n=2 animals; *Lmpt* OE Leg fiber I, n=1 animals; *Lmpt* OE Leg fiber II, n=2 animals; *Lmpt* OE Leg fiber III, n=2 animals). Each point represents value for each animal dataset. Bars represent mean ± SD. Significance determined as p < 0.05 from one way ANOVA with Tukey's (*, p ≤ 0.05; **,p ≤ 0.01; ***,p ≤ 0.001; ****, p ≤ 0.0001; ns, non-significant). (Scale Bars: 5 µm).

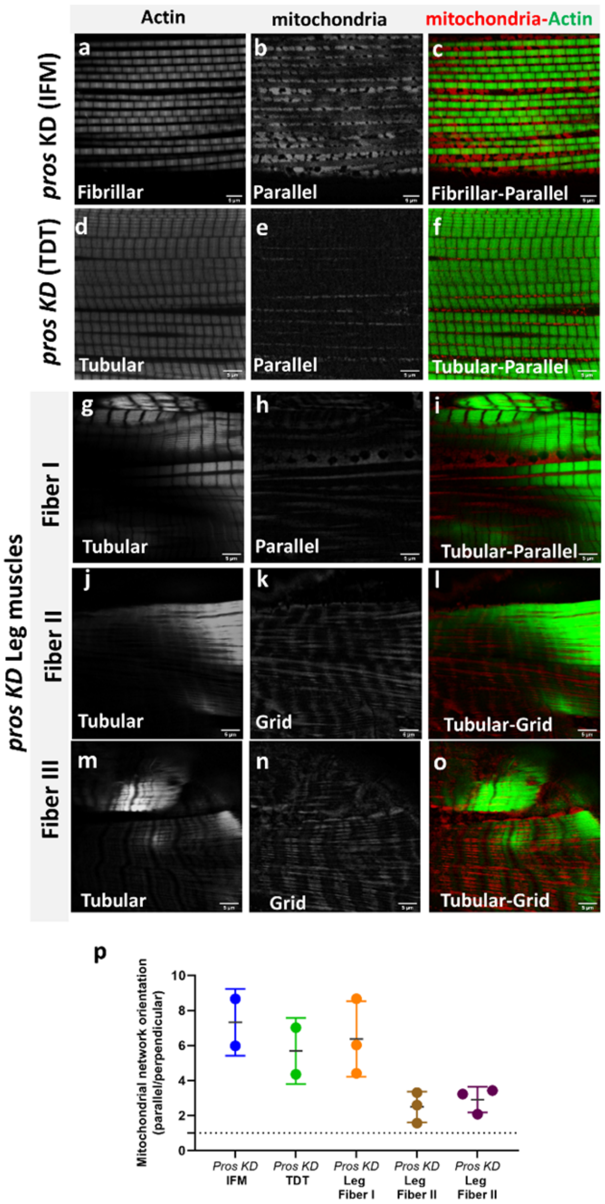

Supplementary Fig. 22. *Pros* knock down in muscles does not affect mitochondrial networks and contractile type in *Drosophila* muscles.

(a, b, c) Adult flight muscles with *pros* KD showing fibrillar myofibrils (ph-TRITC) and parallel mitochondrial networks (mito-gfp). (d, e, f) *pros* KD jump muscles showing tubular contractile type and grid-like

mitochondrial network. (g, h, i) Leg muscle Fiber I with *pros* KD retains parallel mitochondrial networks and tubular muscle fibers. (j, k, l) *pros* KD leg muscle Fiber II and (m, n, o) *pros* KD Fiber III showing grid-like mitochondrial networks and tubular myofibrils (Scale Bars: 5 µm). (p) Quantification of mitochondrial network orientation. Dotted line represents parallel equal to perpendicular(*pros* KD IFM, n= 2; *pros* KD TDT, n=2 animals; *pros* KD Leg fiber I, n=2 animals; *pros* KD Leg fiber II, n=3 animals; *Pros* KD Leg fiber III, n=3 animals). Each point represents value for each dataset. Bars represent mean ± SD. Significance determined as $p < 0.05$ from one way ANOVA with Tukey's (*, $p \leq 0.05$; **,$p \leq 0.01$; ***,$p \leq 0.001$; ****, $p \leq 0.0001$; ns, non-significant). (Scale Bars: 5 µm).

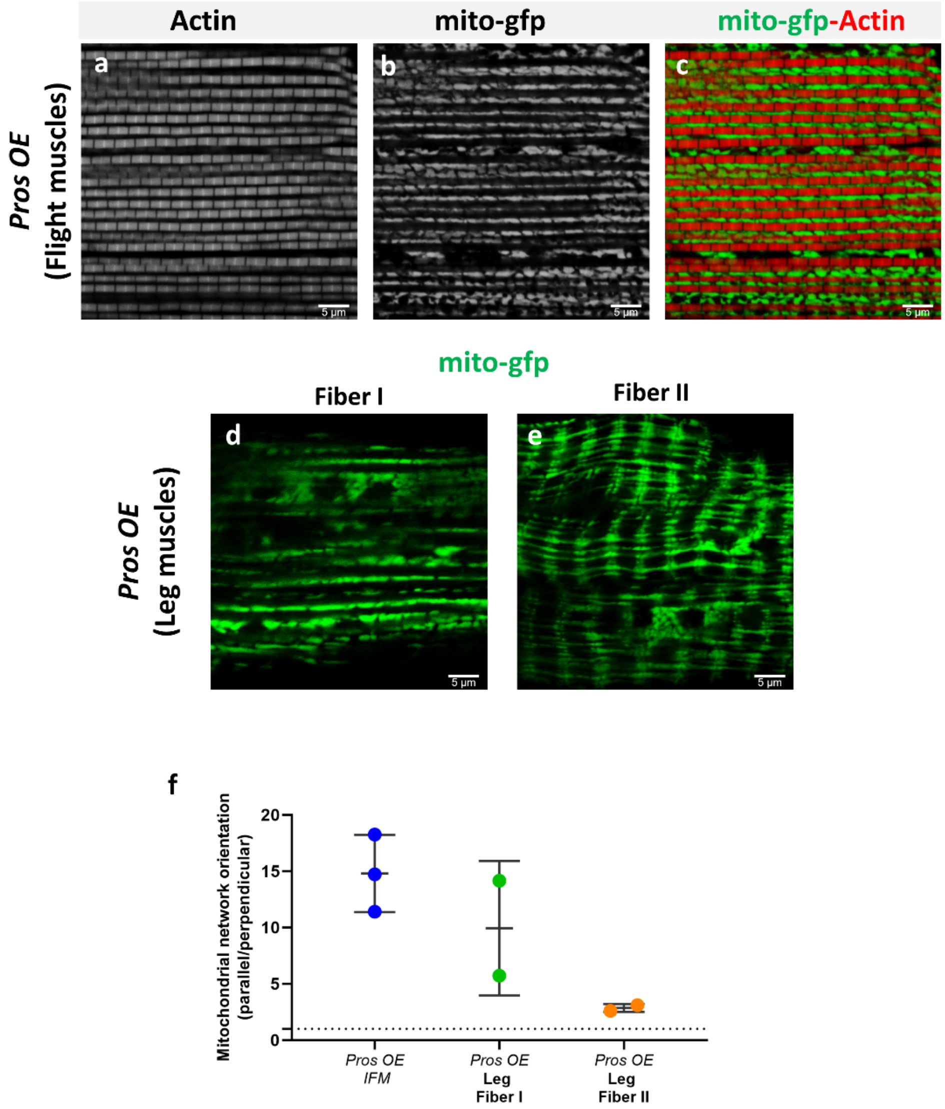

Supplementary Fig. 23. *Pros* overexpression in muscles does not affect mitochondrial networks and contractile type in *Drosophila* muscles.

(a, b, c) Adult flight muscles with *pros* OE showing fibrillar myofibrils (ph-TRITC) and parallel mitochondrial networks (mito-gfp). (d) Leg muscle Fiber I with *pros* OE retains parallel mitochondrial networks (mito-gfp). (e) *pros* OE leg muscle Fiber II showing grid-like mitochondrial networks (Scale Bars: 5 µm). (f) Quantification of mitochondrial network orientation. Dotted line represents parallel equal to

perpendicular (*pros* OE IFM, n= 3 animals; *pros* OE Leg fiber I, n=2 animals; *pros* OE Leg Fiber II, n=2 animals). Each point represents value for each animal dataset. Bars represent mean ± SD. Significance determined as $p < 0.05$ from one way ANOVA with Tukey's (*, $p \leq 0.05$; **,$p \leq 0.01$; ***,$p \leq 0.001$; ****, $p \leq 0.0001$; ns, non-significant).(Scale Bars: 5 µm).

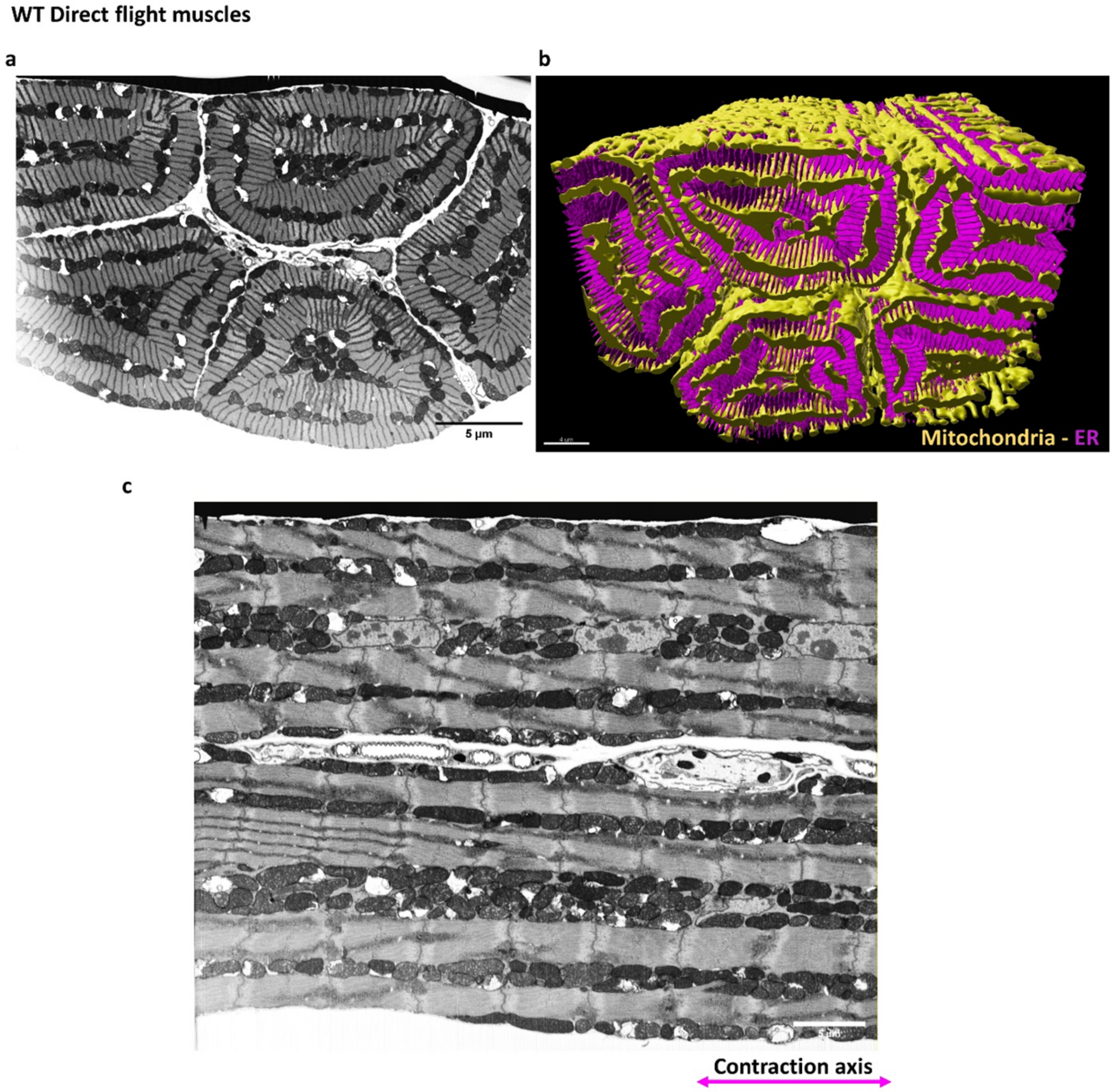

Supplementary Fig. 24. Focused Ion bean scanning electron microscopy (FIB-SEM) images of *Drosophila* direct flight muscles

(a) Transverse view of mitochondrial organization in wildtype direct flight muscles. (b) Representative 3D rendering of electron microscopic images of mitochondrial arrangement (yellow) and ER (magenta) in wildtype direct flight muscles showing mitochondrial networks arranged in sheets parallel to the tubular contractile networks. (c) Longitudinal view of mitochondrial organization in wildtype direct flight muscles. (a & c Scale Bars: 5 µm, b, 4 µm).

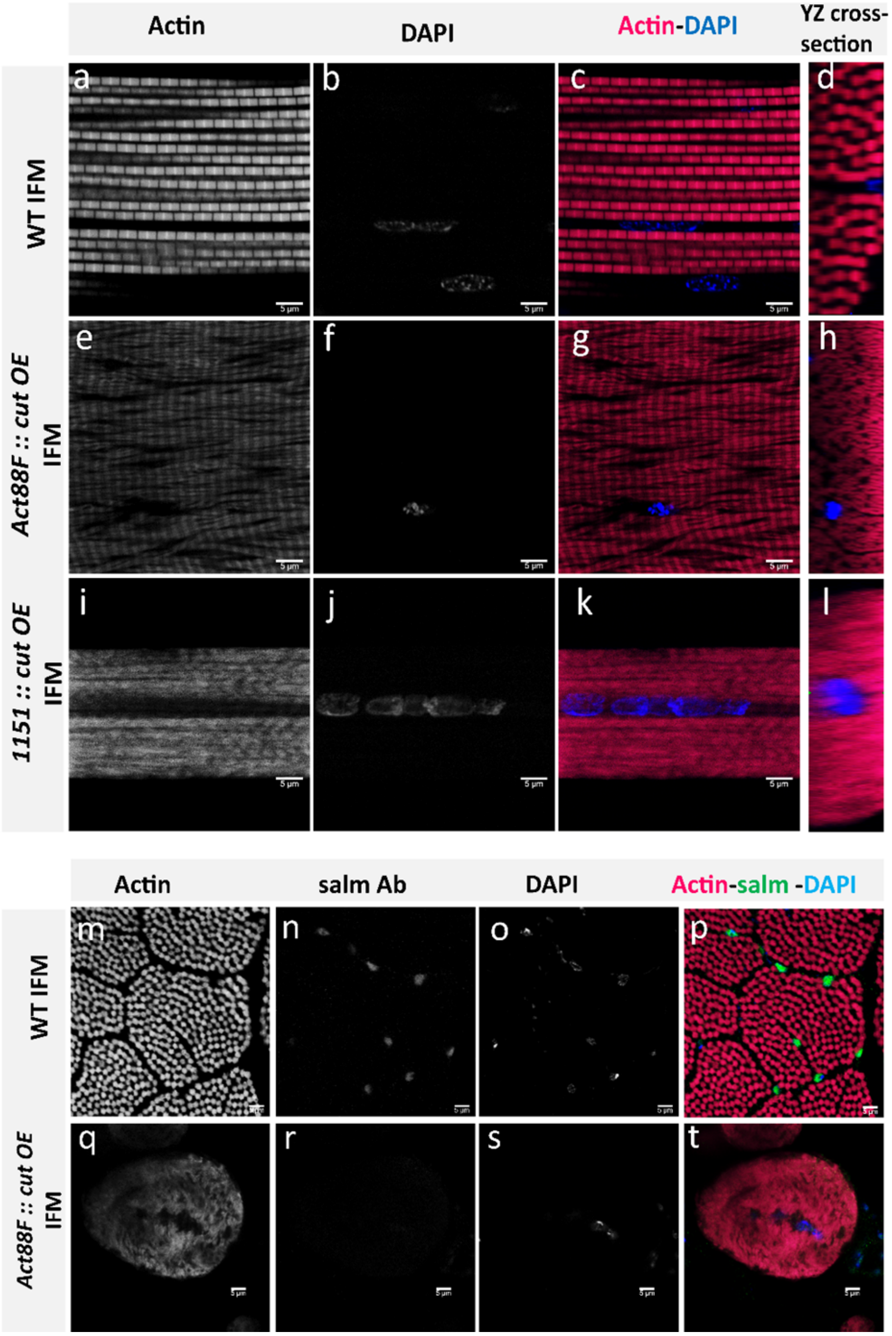

Supplementary Fig. S25. *cut* regulates fiber contractile type in flight muscles.

(a, b, c, d) Adult wild-type flight muscles (fibrillar) stained for F-actin (phTRITC), Nucleus (DAPI). (e, f, g, h) salm KD muscle fiber stained for muscles showing tubular muscle type (Scale Bars: 100 µm). (c, d, e) The overexpression of *cut with Act88F-Gal4* in flight muscles results in fiber conversion to tubular muscle

type. (I, j, k, l) Overexpression of *cut with 1151-Gal4* in flight muscles results in converts to tubular muscle type.(m, n, o, p) Wildtype flight muscles (IFM), stained for F-actin (phTRITC), nuclei (DAPI), and salm antibody. (q, r, s, t) *cut OE (Act88F-Gal4:: cut OE)* IFM showing decreased expression of salm in nuclei (DAPI). (Scale Bars: 5 µm for all).

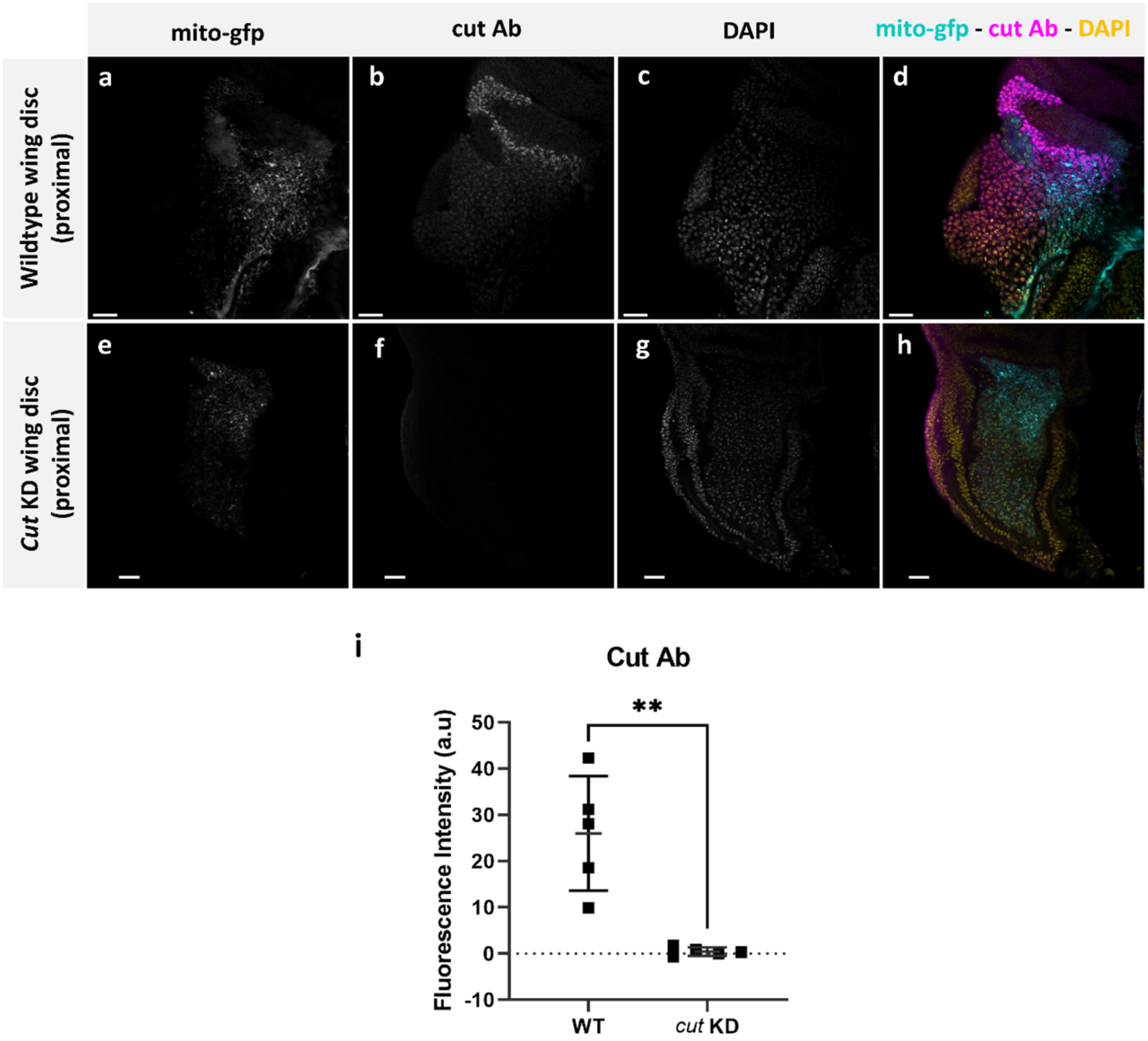

Supplementary Fig. S26. cut expression in wildtype and *cut* knock down wing imaginal discs.

(a-d) Wildtype wing imaginal discs stained for mitochondria (mito-gfp), Cut antibody, and nuclei (DAPI). (e-h) *cut* KD wing imaginal disc showing decreased Cut expression. (i) Quantification of fluorescence intensity of cut antibody staining (WT, n=5 and cut KD, n=5). Each point represents value for each animal dataset. Bars represent mean ± SD. Significance determined as $p < 0.05$ from unpaired t-test with Welch's correction (*, $p \leq 0.05$; **,$p \leq 0.01$; ***,$p \leq 0.001$; ****, $p \leq 0.0001$; ns, non-significant). (Scale Bars: 20 µm for all).

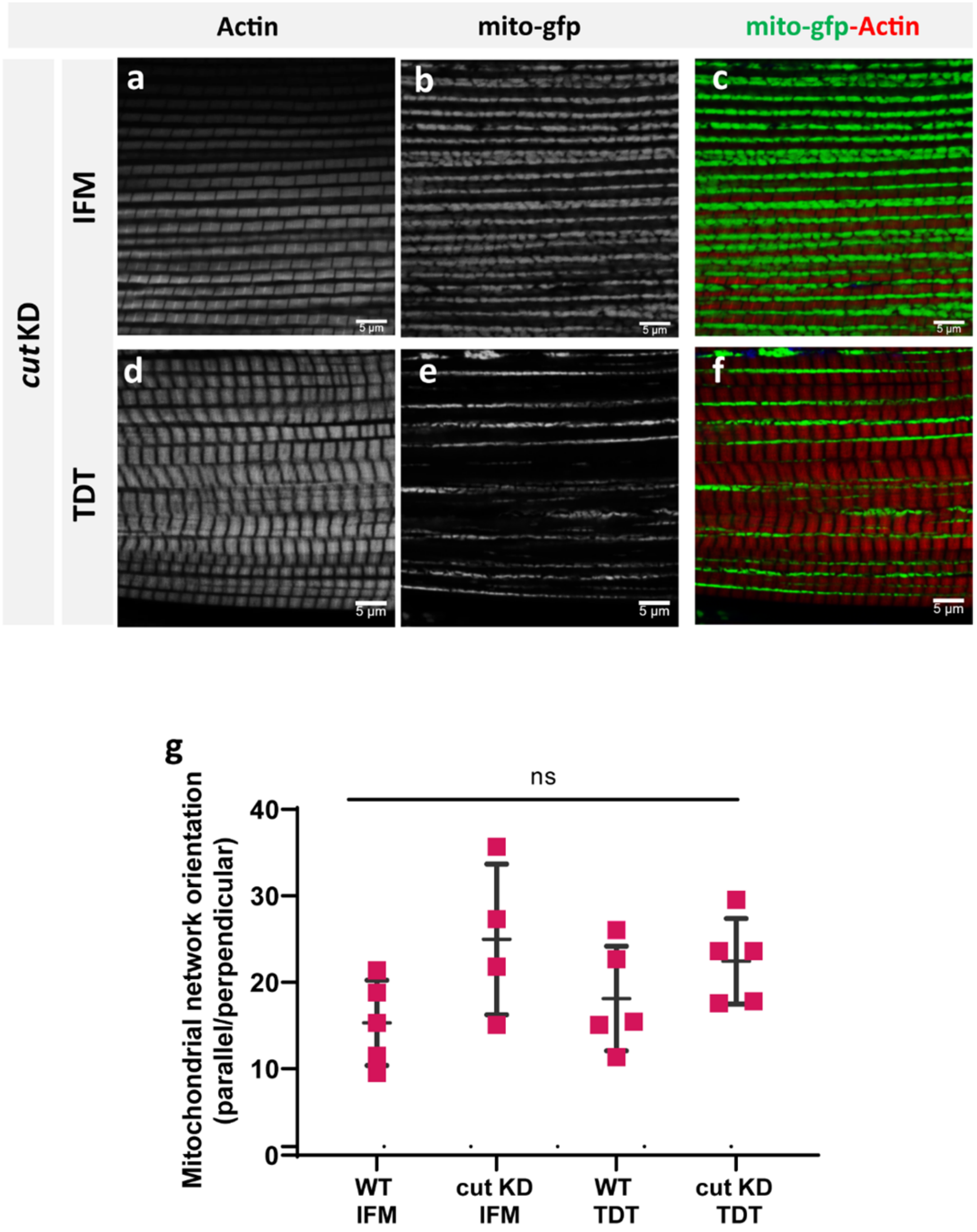

Supplementary Fig. S27. *cut* knock down does not affect flight muscles and jump muscles.

(a, b, c) *cut* KD flight muscles (IFMs) showing parallel aligned mitochondria (mito-gfp) that are large, tube-like, and packed between fibrillar myofibrils (phTRITC). (d, e, f) *cut* KD jump muscles show mitochondria that are thin and elongated arranged in parallel mitochondrial networks between tubular fibers (Scale

Bars: 5 µm). (g) Quantification of mitochondrial network orientation. Dotted line represents parallel equal to perpendicular (WT-IFM, *n=5* animals; *cut KD*-IFM, *n=4* animals; WT-TDT, *n=5* animals; *cut KD*- TDT, *n=5 animals*). Each point represents value for each single animals dataset. Bars represent mean ± SD. Significance determined as $p < 0.05$ from one way ANOVA with Tukey's (*, $p \leq 0.05$; **,$p \leq 0.01$; ***,$p \leq 0.001$; ****, $p \leq 0.0001$; ns, non-significant).

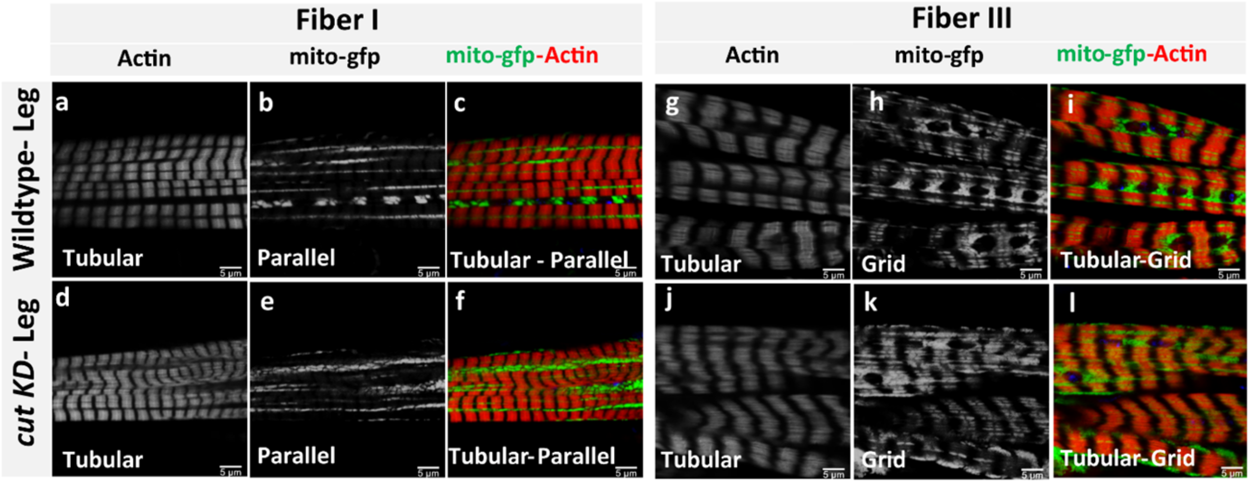

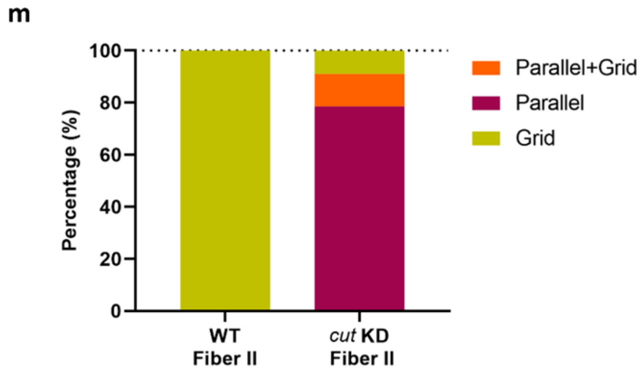

Supplementary Fig. S28. *cut* regulates conversion of mitochondrial networks in Fiber II of *Drosophila* leg muscles.

(a, b, c) Fiber I of wildtype leg muscle showing parallel mitochondrial networks (mito-gfp) and tubular muscle fiber (phTRITC). (d, e, f) *cut* KD Fiber I of leg muscle showing parallel mitochondrial networks GFP) and tubular muscle fiber similar to wildtype Fiber I. (g, h, i) Fiber III of wildtype leg muscle showing grid-like mitochondrial networks and tubular muscle fiber. (j, k, l) *cut* KD Fiber III showing grid-like mitochondrial networks and tubular muscle fiber similar to wildtype Fiber III (Scale Bars: 5 µm). (m) Quantification of percentage of Fibers II exhibiting parallel, grid-like, and parallel as well as grid-like mitochondrial networks in wildtype and *cut* KD Fiber II of leg muscles (WT-Fiber II, *n=7* animal; *cut* KD-Fiber II, *n=14* animals).

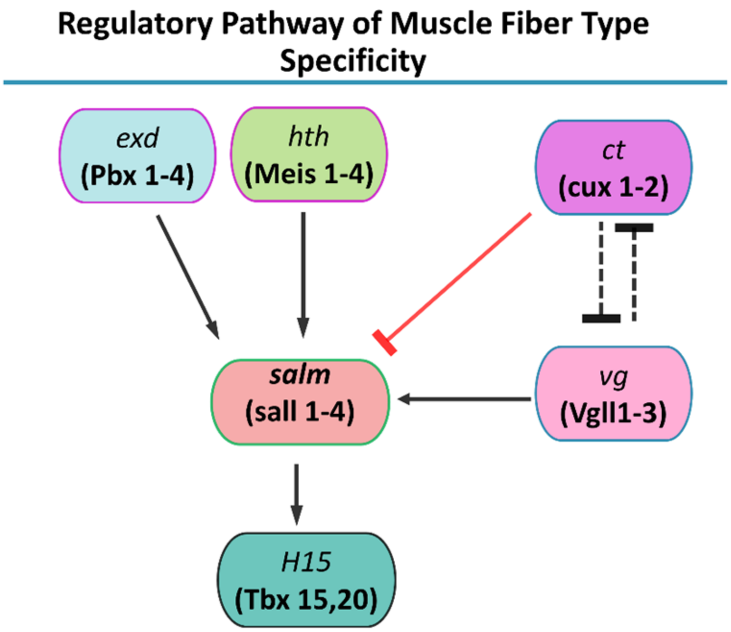

Supplementary Fig. S29. Muscle fiber type specification pathway in *Drosophila*.

Evolutionarily conserved general regulatory pathway of muscle fiber type specificity in Drosophila. Parentheses show mammalian orthologs to Drosophila genes.
