# Supplementary material for: Identification of evolutionarily conserved regulators of muscle mitochondrial network organization
Source: Nat Commun. 2022 Nov 4;13:6622. doi: 10.1038/s41467-022-34445-9 (PMC9636386; doi:10.1038/s41467-022-34445-9)
Supplement: Supplementary file 3 — Description of Additional Supplementary Files [file 41467_2022_34445_MOESM3_ESM.pdf]

Description of Additional Supplementary Files

File Name: Supplementary Data 1
Description: Abundances for 3869 proteins in wild type flight, jump, and leg muscles, *salm* KD flight muscles, and *salm* OE leg muscles.

File Name: Supplementary Data 2
Description: 142 candidate proteins associated with contractile type, mitochondrial network configuration, and/or *salm* expression.

File Name: Supplementary Movie 1
Description: 3D rendering and rotation of the *Drosophila* fibrillar flight muscles (DLMs), bigger parallel mitochondria (Cyan Blue), and myofibrils (Purple).

File Name: Supplementary Movie 2
Description: 3D rendering and rotation of the *Drosophila* jump muscles (TDT), parallel mitochondria (Cyan Blue), and myofibrils (Purple).

File Name: Supplementary Movie 3
Description: 3D rendering and rotation of the *Drosophila* leg coxa muscle Fiber I, parallel mitochondria (Cyan Blue), and myofibrils (Purple).

File Name: Supplementary Movie 4
Description: 3D rendering and rotation of the *Drosophila* leg coxa muscle Fiber II, grid-like mitochondria (Cyan Blue), and myofibrils (Purple).

File Name: Supplementary Movie 5
Description: 3D rendering and rotation of the *Drosophila* leg coxa muscle Fiber III, mitochondria (Cyan Blue), and myofibrils (Purple).

File Name: Supplementary Movie 6
Description: 3D rendering and rotation of the *H15* KD *Drosophila* jump muscle (TDT), mitochondria (Cyan Blue), and myofibrils (Purple).

File Name: Supplementary Movie 7
Description: 3D rendering and rotation of the *H15* KD *Drosophila* Leg coxa muscle fiber I grid-like mitochondria (Cyan Blue).

File Name: Supplementary Movie 8
Description: 3D rendering and rotation of the *cut* KD *Drosophila* coxa leg fiber II, parallel mitochondria (Cyan Blue), and myofibrils (Purple).
