# Supplementary material for: Identification of evolutionarily conserved regulators of muscle mitochondrial network organization
Source: Nat Commun. 2022 Nov 4;13:6622. doi: 10.1038/s41467-022-34445-9 (PMC9636386; doi:10.1038/s41467-022-34445-9)
Supplement: Supplementary file 14 — Reporting Summary [file 41467_2022_34445_MOESM14_ESM.pdf]

nature portfolio

Corresponding author(s): Glancy, Brian

Last updated by author(s): Oct 12, 2022

Reporting Summary

Nature Portfolio wishes to improve the reproducibility of the work that we publish. This form provides structure for consistency and transparency in reporting. For further information on Nature Portfolio policies, see our Editorial Policies and the Editorial Policy Checklist.

Statistics

For all statistical analyses, confirm that the following items are present in the figure legend, table legend, main text, or Methods section.

n/a	Confirmed	
☐	☒	The exact sample size (*n*) for each experimental group/condition, given as a discrete number and unit of measurement
☐	☒	A statement on whether measurements were taken from distinct samples or whether the same sample was measured repeatedly
☐	☒	The statistical test(s) used AND whether they are one- or two-sided<br>*Only common tests should be described solely by name; describe more complex techniques in the Methods section.*
☐	☒	A description of all covariates tested
☐	☒	A description of any assumptions or corrections, such as tests of normality and adjustment for multiple comparisons
☐	☒	A full description of the statistical parameters including central tendency (e.g. means) or other basic estimates (e.g. regression coefficient) AND variation (e.g. standard deviation) or associated estimates of uncertainty (e.g. confidence intervals)
☐	☒	For null hypothesis testing, the test statistic (e.g. *F*, *t*, *r*) with confidence intervals, effect sizes, degrees of freedom and *P* value noted<br>*Give P values as exact values whenever suitable.*
☒	☐	For Bayesian analysis, information on the choice of priors and Markov chain Monte Carlo settings
☒	☐	For hierarchical and complex designs, identification of the appropriate level for tests and full reporting of outcomes
☒	☐	Estimates of effect sizes (e.g. Cohen's *d*, Pearson's *r*), indicating how they were calculated

Our web collection on statistics for biologists contains articles on many of the points above.

Software and code

Policy information about availability of computer code

Data collection	ZEISS Crossbeam 540 with ZEISS Atlas 5 software, QuantStudio 3 Real-Time PCR System
Data analysis	ImageJ v1.53p, Ilastik 1.3.3post2, Imaris 9.7.0, Excel 2016, Prism 9.0.0, ZEN 3.2.0.115, ProteomeDiscoverer 2.3, Mascot 2.6.2

For manuscripts utilizing custom algorithms or software that are central to the research but not yet described in published literature, software must be made available to editors and reviewers. We strongly encourage code deposition in a community repository (e.g. GitHub). See the Nature Portfolio guidelines for submitting code & software for further information.

Data

Policy information about availability of data

All manuscripts must include a data availability statement. This statement should provide the following information, where applicable:

- Accession codes, unique identifiers, or web links for publicly available datasets
- A description of any restrictions on data availability
- For clinical datasets or third party data, please ensure that the statement adheres to our policy

Protein abundance data for the proteomics screen has been provided as supplemental dataset 1. Raw mass spectrometry data has been uploaded to MassIVE117 repository (MSV000088173). All raw image data used in this work is available upon reasonable request. Data files were searched against Translated EMBL (TrEMBL) Drosophila melanogaster protein sequence database (uniprot.org)

Human research participants

Policy information about studies involving human research participants and Sex and Gender in Research.

Reporting on sex and gender	N/A
Population characteristics	N/A
Recruitment	N/A
Ethics oversight	N/A

Note that full information on the approval of the study protocol must also be provided in the manuscript.

Field-specific reporting

Please select the one below that is the best fit for your research. If you are not sure, read the appropriate sections before making your selection.

☒ Life sciences ☐ Behavioural & social sciences ☐ Ecological, evolutionary & environmental sciences

For a reference copy of the document with all sections, see nature.com/documents/nr-reporting-summary-flat.pdf

Life sciences study design

All studies must disclose on these points even when the disclosure is negative.

Sample size	Sample sizes were chosen based on previous studies using mitochondrial and contractile morphology measurements in Bleck et al. Nat Comms, 2018 and Willingham et al. Nat Comms, 2020.
Data exclusions	No data were excluded
Replication	All studies were successfully repeated in at least three biological replicates
Randomization	No randomization was performed due to the clear phenotype differences among muscle types.
Blinding	No blinding was performed due to the clear phenotype differences among muscle types.

Reporting for specific materials, systems and methods

We require information from authors about some types of materials, experimental systems and methods used in many studies. Here, indicate whether each material, system or method listed is relevant to your study. If you are not sure if a list item applies to your research, read the appropriate section before selecting a response.

Materials & experimental systems

n/a	Involved in the study	
☐	☒	Antibodies
☒	☐	Eukaryotic cell lines
☒	☐	Palaeontology and archaeology
☐	☒	Animals and other organisms
☒	☐	Clinical data
☒	☐	Dual use research of concern

Methods

n/a	Involved in the study	
☒	☐	ChIP-seq
☒	☐	Flow cytometry
☒	☐	MRI-based neuroimaging

Antibodies

Antibodies used	Rabbit anti-salm-1 (1:500, gift from Dr. Tiffany Cook,110), Rabbit anti-nmr1 (H15) (1:200, gift from Dr. James B. Skeath,111), Mouse anti-cut (1:20, 2B10, DHSB), Alexa Fluor 594-labeled Goat anti-Mouse IgG (1:500, Cat# A-11032) and Alexa-Fluor-488-labeled anti-rabbit IgG (1:500, Cat# A32731, Thermofisher, USA).
Validation	All primary antibodies (salm, H15, cut) were validated in this work by the loss of signal (Sup Fig 6o, Sup Fig 18f, Sup Fig 26f, respectively) when each gene was knocked down.

Animals and other research organisms

Policy information about studies involving animals; ARRIVE guidelines recommended for reporting animal research, and Sex and Gender in Research

Laboratory animals	1-4 day old flies were used for all studies. W1118 were used as controls and respective genetic backgrounds. Mef2- Gal4 (III) was used to drive muscle specific gene knockdown and over expression of respective genes. Tub-Gal80ts; Mef2 Gal4 used for ectopic expression of salm in muscles. UAS-mito-GFP (II chromosome BS# 8442) was used for mitochondrial network visualization. UAS-mito-mcherry (III) was used for visualization of the outer mitochondrial membrane. UAS- H15 RNAi trip lines were used for muscle specific knock down of H15 gene. UAS-mito-mcherry (BS# 66533), Mef2-Gal4 (BS# 27390), Act88F-Gal4 (III, BS# 38461), 1151-Gal4105,106 (I, gift from Dr. Upendra Nongthomba) UAS-H15 RNAi (V28415), UAS-Drp1RNAi (BS# 51483), Marf RNAi (BS# 55189), UAS- salm (Dr. Frank Schnorrer), UAS-salm RNAi (V101052), UAS-Miro RNAi (V106683), UAS-Fis1 RNAi (BS# 63027), UAS-cut RNAi (BS# 33967, #29625), UAS-H15/nmr1 (Dr. Rolf Bodmer), UAS- cut107 (gift from Dr. Yuh Nung Jan, UCSF, USA), cut-OE (TOE.GS00041) (BS# 67524), UAS-Cas9.P2; Mef2-GAL4 /TM6B,(BS# 67075), UAS-dCas9/cyO; Mef2-GAL4 (BS# 67041), H15-OE (BS# 78722)108 , UAS-RFP.KDEL (BS# 30910, # 30909) (Dr. Richa Rikhy),Zasp52MI02908-mCherry109 (Dr. Frieder Schöck). UAS-pros ORF (F004799, FlyORF), UAS- pros RNAi (BS# 26745), UAS-Lmpt.ORF.3xHA. (F001889), UAS-Lmpt RNAi (v105170, 100716). All other stocks were requested or obtained from the VDRC (Vienna) Drosophila stock center, Bloomington (BS#) Drosophila stock center or FLYORF (Zurich) Drosophila stock center. All chromosomes and gene symbols are as mentioned in Flybase (http://flybase.org).
Wild animals	No wild animals were used
Reporting on sex	Both males and females were used and grouped together due an observed lack of sex differences in mitochondrial network configuration in wild type muscles.
Field-collected samples	No field collected samples were used
Ethics oversight	No ethical approvals are required for Drosophila studies

Note that full information on the approval of the study protocol must also be provided in the manuscript.
